# Response to multigenerational graphene oxide exposure in acheta domesticus strains selected for longevity

**DOI:** 10.1038/s41598-026-37623-7

**Published:** 2026-01-30

**Authors:** Barbara Flasz, Agnieszka Babczyńska, Monika Tarnawska, Amrendra K. Ajay, Andrzej Kędziorski, Łukasz Napora-Rutkowski, Ewa Świerczek, Katarzyna Rozpędek, Maria Augustyniak

**Affiliations:** 1 Institute of Biology, Biotechnology and Environmental Protection, University of Silesia in Katowice, Katowice, Poland; 2 Department of Medicine, Division of Renal Medicine, Brigham and Women’s Hospital, Harvard Medical School, Boston, MA 02115 USA; 3 Polish Academy of Sciences, Institute of Ichthyobiology and Aquaculture in Gołysz, Chybie, 43–520 Poland

**Keywords:** Graphene oxide, DNA damage, Mitochondrial potential, Apoptosis, Multigenerational effects, Epigenetics, Developmental biology, Ecology, Ecology, Evolution

## Abstract

The development of new nanotechnologies and their use in everyday life always carries the risk of environmental hazards and consequences for human health. Among them, graphene oxide (GO) is a promising material. Due to its excellent physicochemical properties, GO is attractive not only for industrial applications but also in medicine. There is still a lack of sufficient reports on the long-term effects of GO on organisms, including studies of a multigenerational nature. We investigated the health status of two strains of *Acheta domesticus*: the wild type and the long-lived. The strains were exposed to GO for five generations and a sixth recovery generation. We investigated parameters that may indirectly explain the mechanisms involved in transmitting the informational pattern of the stress response to subsequent generations: DNA stability, mitochondrial potential, apoptosis, and autophagy. GO intoxication induced multilevel cellular responses in five subsequent generations. GO cessation in recovery F5 acted as a new stressor. Across five generations, variation in the response to GO was observed. GO is most likely responsible for changes that persist over generations. We believe that epigenetic inheritance is a likely mechanism underlying the multigenerational adaptation observed in GO-exposed insects, and future research should aim to elucidate this phenomenon in more detail.

**Supplementary Information:**

The online version contains supplementary material available at 10.1038/s41598-026-37623-7.

## Introduction

Graphene oxide is a promising nanoparticle that has gained attention in different fields of industry and biomedicine^1–3^. The development of new nanotechnologies and their application in everyday life carries the risk of environmental hazards and adverse health consequences. Therefore, research has been ongoing for years to investigate the mechanisms of toxicity of various nanoparticles, including graphene oxide^4–6^. The short-term effects of GO are relatively well-documented by numerous studies^7–10^. Unfortunately, there is still a lack of sufficient reports on the long-term effects of GO on organisms, including studies of a multigenerational and transgenerational nature. One study investigated the impact of six-generation intoxication with GO, which caused damage to neuron development and reproductive systems in the nematode *Caenorhabditis elegans*^11^. In another study, *Daphnia magna* was examined to determine how GO with different surface chemical modifications affected two generations of this model aquatic species. The varying impacts of the six reduced GO nanocomposites on *D. magna* were primarily influenced by their specific surface area and the type of metal incorporated. Moreover, the findings provide strong evidence for maternal effects, which contributed to increased susceptibility of the offspring to toxic stress, along with reduced survival and reproductive capacity^12^. The transgenerational impact and influence of maternal exposure were investigated in Japanese medaka embryos, where long-term exposure of embryos and adults resulted in lower fecundity. Moreover, the effect was transmitted to the next F1 and F2 generations^13^. The study of maternal effects, or long-term exposure to nanoparticles across generations, raises essential questions about the mechanisms involved in transmitting information about stress-coping strategies to offspring and how they can adapt to a constantly present stressor. What mechanisms are responsible for passing information about the stressor to subsequent generations? One proposed mechanism is epigenetic modification^14^. Epigenetics occurs when dynamic changes to DNA affect gene expression apart from alterations to the underlying sequence^15^. The changes may involve DNA methylation, histone modifications, and non-coding RNA regulation^16^. In our previous work, we investigated the multigenerational effects of GO in low concentrations on the invertebrate *Acheta domesticus* (Orthoptera: Gryllidae). We focused on DNA stability, measured as the level of apurinic/apyrimidinic sites, the share of 8-hydroxy-2’-deoxyguanosine, and global DNA methylation as an epigenetic marker^17^. Although the pattern of global DNA methylation was comparable among the five intoxicated generations, we suggest that other epigenetic mechanisms are also involved.

Our team’s previous studies and emerging conclusions on the toxicity of graphene oxide (GO) have emphasized the necessity of conducting studies on the effects of GO on subsequent generations, even in cases where the toxic material has been removed^9,17–20^. Transgenerational and multigenerational exposure studies are particularly important for short-lived organisms, such as insects, which are the most numerous taxonomic group among animals on Earth and play a crucial role in the proper functioning of ecosystems. Pollution of air, soil, or water due to accidental release can have significant effects on subsequent generations living in a contaminated environment. Short-lived animals can serve as an interesting model, allowing us to evaluate not only the direct impact of GO on exposed individuals but also its potential cumulative and inherited effects on subsequent generations. The use of strains differing in longevity is justified because it may reveal different strategies of response to stress factors. In this work, we investigated the health status of two strains of *Acheta domesticus*, which differ in ontogenetic development: the wild type and the long-lived. These strains were exposed to GO for five generations and a sixth recovery generation. In this project, we focused on parameters that may indirectly explain the mechanisms involved in transmitting the informational pattern of the stress response to subsequent generations. We analyzed how DNA stability, mitochondrial potential, apoptosis, and autophagy changed across six studied generations. The study aimed to determine whether information on effectively responding to a continuously present stressor is transmitted to subsequent generations and whether this transmission contributes to improved cellular parameters and the establishment of a new homeostatic balance.

## Materials and methods

### Graphene oxide characteristics

Graphene oxide was produced as a water suspension at 10 mg·mL^−1^ (Nanografi, Germany). The product was sonicated in an ultrasonic bath and diluted to 20 µg·mL^−1^. The GO samples for scanning electron microscopy (SEM) and atomic force microscopy (AFM) visualization were prepared. The SEM (Quanta FEG 250, FEI, Oregon, USA, accelerating voltage of 30 kV in high vacuum mode), AFM Agilent 5500 (Agilent Technologies, CA, USA, tapping mode), and Litesizer 500 (the zeta potential, Anton Paar, Graz, Austria) were used to measure GO flakes’ morphology. The zeta potential values were calculated using the Smoluchowski equation.

**Fig. 1:**
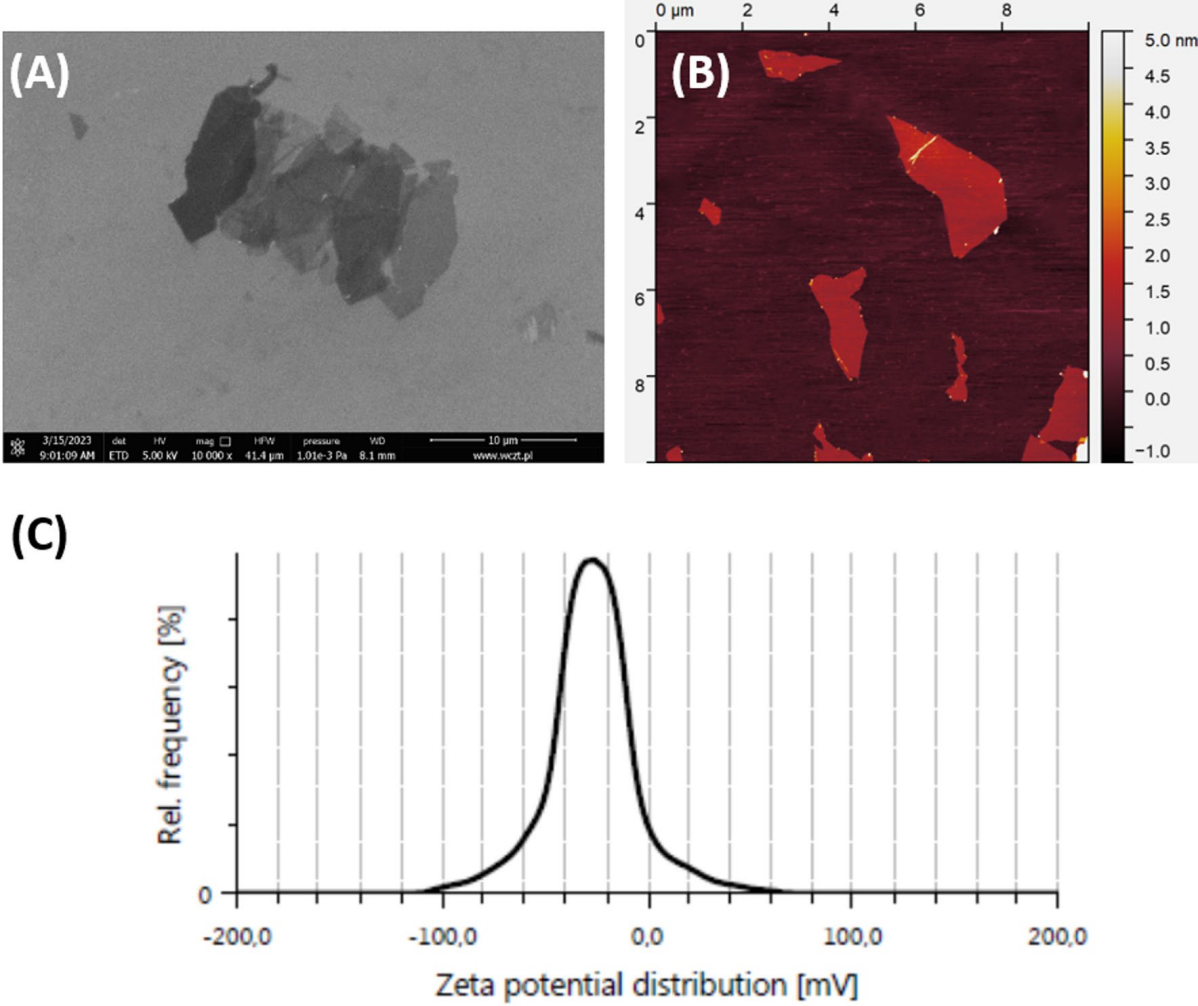
Image of graphene oxide (**A**) SEM - scale bar 10 μm; (**B, C**) AFM, and zeta potential of GO dispersion in water.

The detailed characteristics of GO can be found in^21^, where the GO sample was marked as S3. Briefly, the AFM and SEM visualization revealed GO single layers with a thickness of approximately 1.0 nm, and the average area of a flake was 2 μm² (Fig. 1). The zeta potential was approximately − 30 mV.

### Characteristics of the species

*Acheta domesticus* (*Gryllidae*,* Orthoptera*,* Insecta*) is a found worldwide insect with nutritional relevance. Due to its numerous advantages, including low breeding costs, high reproductive success, and straightforward tissue collection procedures, it is proposed as a promising model organism^22^. House cricket is a hemimetabolous insect. The life cycle lasts approximately three months, comprising eight nymphal stages. As it is widely distributed across different ecosystems, the invertebrate is a suitable model for studying environmental pollution in both industrial and rural sites. *A. domesticus* has been used at the University of Silesia in Katowice since 2001, and two unique strains are, in effect, being maintained through continuous selective breeding: wild-type (H) and long-lived (D). The strains differ in ontogenetic development. The D strain includes several cohorts selected by postponed reproduction. The H strain is not selected for longevity, and it is allowed to reproduce regardless of the age of the adults. Adults older than 35 days may reproduce in the D strain and are separated at final molting. The D strain selection resulted in decreased larval mortality (50% survival time is extended by 25–46% in particular generations) and extension of adult life. Data from 30 adult cohorts of two subsequent generations revealed a mean survival time of 50% (since final molting) to be 57.6 ± 17.8 and 60.1 ± 20.6 days for males and females, respectively. The mean 50% survival time H strain cohorts (*n* = 27) is 22.2 ± 5.17 (males) and 22.6 ± 5.34 days (females)^23^.

### Graphene oxide and control food preparation

The insects were fed standard artificial rabbit food (Kanisan Q, Sano, Poland). The food pellet was ground and mixed with GO suspension, which was diluted in ultrapure water to obtain the final GO concentration as follows: lower (L) at 0.02 µg∙g^−1^ of food, and higher (H) at 0.2 µg∙g^−1^ of food. Then, the food was dried (24 h, 45 °C), sterilized with the UV lamp (24 h), and kept in dry glass jars in the dark. Control (C) food was prepared using the same method, without the addition of GO.

### Experimental model

The insects were kept in a breeding room with optimal conditions for growth and reproduction (temperature 28.8 ± 0.88 °C, photoperiod L: D 12:12, and humidity 20–45%). One week after hatching, nymphs were in the early nymphal stage (approximately second instar), actively feeding and growing but not yet developing wing buds or reproductive organs. Nymphs, one week old, in the first generation (F0), were divided into experimental groups. Six experimental groups were fed with water and food *ad libitum*. Three groups were designed for the H strain: control (HC), lower GO (HL), and higher GO (HH). Similarly, three groups were designed for the D strain: control (DC), lower GO (DL), and higher GO (DH).

In every generation (F0-F5), when *A. domesticus* males and females reached maturity, they were allowed to reproduce within their experimental groups, and the eggs were protected under optimal conditions for the hatching process, ensuring the continuity of the next generation and breeding. For survival monitoring purposes, in each group and every generation, the culture was initiated with approximately 1,500 first-instar larvae, placed in three rearing containers (46 cm × 31 cm × 17.5 cm; 500 individuals per container). As the insects developed, populations were redistributed as needed, depending on growth and mortality levels, to adjust density and reduce crowding.

The culture was maintained continuously. For breeding, an adequate number of insects was secured, resulting in the successful hatching and development of individuals into adulthood. Among the adult insects, enough individuals were secured to carry out the planned biochemical analyses. The lack of data for strain D, group H, F4 was not due to insect mortality, but resulted from technical issues. On the day of sampling, the survival rate in the obtained generations was as follows (%; mean ± SD): F0 53.2 ± 9.5; F1 60.9 ± 8.2; F2 55.1 ± 12.1; F3 55.7 ± 5.3; F4 53.5 ± 6.2; F5 59.8 ± 10.1.

For biochemical and cellular analyses, separate plastic boxes (28 cm × 20 cm × 16 cm; 20 individuals each) were established in three replicates for every group in each generation. Once the insects reached adulthood, individuals were randomly sampled from these boxes (*n* = 5 per experimental group) and subjected to the assays described in this study. The remaining insects were used to determine other biochemical parameters.

In the last recovery generation (F5), insects were no longer fed with GO.

### Sample preparation and measurement of selected parameters

#### Sample: tissue preparation for DNA damage, cell viability, mitochondrial transmembrane potential, autophagy, apoptosis

The selected parameters were measured with flow cytometry. The digestive tract was isolated from insects and placed on ice in 400 µL of PBS (phosphate-buffered saline, pH 7.4, 0.1 M). Samples were homogenized using a microtube homogenizer (Minilys, Bertin Technologies) and then filtered to obtain the cell suspension required for flow cytometry. The samples from experimental groups were analyzed using the Cytek Guava Muse cell analyzer flow cytometer (Cytek Biosciences, Fremont, CA, USA).

#### Flow cytometry: DNA damage

The DNA damage measured as double-strand breaks (DSB), histone phosphorylation at position Ser139 (H2A.X), and the ATM protein phosphorylation (pATM) that coordinates the DNA repair processes by activating relevant enzymes, was assessed with Muse Multi-Color DNA Damage Kit (Cytek Biosciences, Fremont, CA, USA; MCH200107).

#### Flow cytometry: mitochondrial transmembrane potential and cell viability

The Muse MitoPotential Kit (Cytek Biosciences, Fremont, CA, USA; MCH100110) was used to measure the viability of cells and changes in mitochondrial transmembrane potential, which can be a marker of early apoptosis. Following the protocol, the fluorescent-based analysis of mitochondrial dysfunction was estimated. Four populations of cells were distinguished: live cells with intact mitochondrial membrane (live), dead cells with intact mitochondrial membrane (dead), live cells with depolarized mitochondrial membrane (depolarized, live), and dead cells with depolarized mitochondrial membrane (depolarized, dead).

#### Flow cytometry: autophagy

The Muse Autophagy LC3-antibody Based Kit (Cytek Biosciences, Fremont, CA, USA; MCH200109) enables the estimation of mean autophagy intensity via antibody-based detection of autophagosomal LC3 protein. According to the manufacturer’s protocol, the control sample was measured for all experimental groups, serving as a reference. For autophagy intensity measurements, cell suspensions from midgut tissues of control individuals were pooled within a strain for each generation separately.

#### Flow cytometry: apoptosis

The Muse Annexin V & Dead Cell Kit (Cytek Biosciences, Fremont, CA, USA; MCH100105) was used to measure apoptosis. Annexin V was used in the protocol due to its high affinity for phosphatidylserine (PS). PS externalization to the outer surface of the bilipid layer is characteristic of apoptosis, and Annexin binds to PS located on the cell surface. The kit also includes propidium iodide to mark dead cells. The assay measured early apoptotic, late apoptotic, and total apoptotic cells.

### Statistical analysis

All statistical analyses were performed using permutation-based approaches to accommodate small sample sizes and potential violations of normality and homoscedasticity. For each parameter, a univariate permutational ANOVA (PERM-ANOVA) was conducted with Strain, Group, and Generation as fixed factors, including all interaction terms. All models were fitted using the Freedman-Lane permutation strategy with 9999 permutations and Euclidean distances. Because the highest-order interaction (Strain × Group × Generation) was significant for several parameters, two-way PERM-ANOVAs (Strain × Group) were subsequently performed within each generation. Significant interactions were followed by pairwise permutation tests (C vs. L, C vs. H, L vs. H) conducted separately for each strain and generation using the same Freedman-Lane framework. Resulting p-values were corrected for multiple comparisons using the Benjamini-Hochberg false discovery rate (FDR). Visualization of univariate results included mean ± 95% confidence intervals for each parameter. Statistically significant post-hoc differences were presented using letter annotations, where different letters denote significant differences between treatment groups.

To evaluate multivariate treatment effects across all biomarkers jointly, a multivariate PERMANOVA (Euclidean distance; 9999 permutations) was conducted on a z-standardized matrix of all measured variables using a full three-way factorial model (Strain × Group × Generation). Multivariate patterns were visualized using principal component analysis (PCA), which was performed on the same standardized data. Clean PCA plots (centroids only, trajectories across generations) were generated to illustrate temporal dynamics and strain-specific responses. Additionally, a “distance-to-control” metric was computed as the Euclidean distance of each sample to the centroid of the corresponding control group (within each strain and generation) in PCA space, providing a univariate summary of multivariate deviation from baseline. All analyses and visualizations were performed in Python (Python 3.14.0, https://www.python.org/downloads/) using custom scripts based on statsmodels, patsy, numpy, scikit-learn, and matplotlib.

In addition to the permutation-based analyses described above, supplementary parametric analyses were conducted to provide complementary visualization and to facilitate comparison with conventional statistical approaches. For this purpose, all variables were also expressed as relative values normalized to the control group (C = 1) within each strain and generation. Based on these transformed data, classical parametric tests were applied, including one-way ANOVA (Fisher’s test, *p* < 0.05) to assess differences between treatment groups. Furthermore, a multivariate repeated-measures ANOVA was performed with Strain, Group, Generation, and their interactions as fixed factors. Expected marginal means were calculated for strains H and D across generations (F0–F5) to visualize temporal trends and strain-specific responses under the parametric framework. These complementary analyses served solely as supporting material, providing an alternative representation of the data and confirming the general trends observed using the permutation-based approach. All supplementary parametric analyses and visualizations were performed using Statistica software (STATISTICA^®^ 13.3 PL, StatSoft Inc., Poland), and the full results are presented in the Supplementary Material.

The following hypotheses were tested:

H1. DNA damage and/or cell viability and/or mitopotential and/or autophagy and/or apoptosis and gut health parameters are impacted by GO applied in two concentrations, and the effects may vary between generations.

H2. The cessation of GO from the food in generation F5 is expected to restore the baseline levels of the examined health parameters.

H3. Different life histories resulting from selection for longevity may influence the intensity of selected cellular responses to GO.

## Results

### DNA damage

Across all DNA-damage–related biomarkers, responses exhibited substantial temporal variation with multiple treatment-related differences within individual generations; however, the overall Strain × Group × Generation interaction was significant only for Total Damage, while pATM, DSB, and pH2A.X showed non-significant highest-order interactions (Table 1). This indicates that although group differences appeared in specific generations, only Total Damage displayed a consistent multigenerational strain-dependent pattern in response to GO.

**Table 1:** Results of three-way PERMANOVA (Strain × group × Generation) for all measured physiological and cellular parameters. Univariate PERMANOVA models were computed for each variable using Euclidean distances and the Freedman-Lane permutation method with 9999 permutations. Reported are the pseudo-F statistics and permutation-based p-values (p_perm_) for the highest-order interaction term (Strain × group × Generation), together with numerator (d_fnum_) and denominator (d_fden_) degrees of freedom. Significant interactions indicate that the effects of GO treatments differ between strains and change across generations.

Variable	Pseudo-F	*p*_perm_	df_num_	df_den_
pATM	1.515	0.1430	10	132
DSB	1.596	0.1135	10	132
pH2A.X	0.892	0.5574	10	132
Total Damage	2.470	0.0083	10	144
Depolarized/Live cells	5.786	0.0001	10	144
Depolarized/Dead cells	5.890	0.0001	10	144
Total depolarized cells	7.204	0.0001	10	144
Live cells	1.987	0.0360	10	144
Dead cells	6.376	0.0001	10	144
MAI	0.720	0.5964	10	91
Early Apoptotic	2.264	0.0218	10	140
Late Apoptotic	0.827	0.5983	10	140
Total Apoptotic	1.995	0.0453	10	140

#### Ataxia telangiectasia mutated kinase phosphorylation (pATM)

pATM levels showed pronounced temporal variation but no significant global Strain × Group × Generation interaction (three-way PERMANOVA: Pseudo-F = 1.515, pperm = 0.143; Table 1). This indicates that, when all generations are evaluated jointly in a multivariate factorial framework, the treatment-dependent responses of strains H and D were not statistically different across generations. However, generation-specific analyses revealed several local but non-systematic interaction effects (Table 2). Two-way PERMANOVA performed separately for each generation showed that the Strain × Group interaction was marginal in F3 (pperm = 0.0689) and F5 (pperm = 0.0650), whereas all other generations showed clearly non-significant interactions (pperm > 0.59). These results indicate that differences between strains in their response to GO exposure occurred only sporadically and did not follow a consistent generational pattern.

**Table 2:** Strain × group interaction effects across generations (F0–F5) estimated using per-generation two-way PERMANOVA. For each parameter and generation, a univariate PERMANOVA model was computed using the Freedman-Lane method with Euclidean distances and 9999 permutations. Shown are permutation-based p-values (pperm) for the strain × group interaction only, reflecting strain-dependent responses to GO at each generation.

Variable	F0	F1	F2	F3	F4	F5
pATM	0.6682	0.5960	0.7280	0.0689	0.9383	0.0650
DSB	0.4590	0.0555	0.1212	0.0845	0.6389	0.1060
pH2A.X	0.1150	0.6814	0.4232	0.6648	0.0381	0.8358
Total Damage	0.6511	0.0522	0.4965	0.0183	0.6604	0.0243
Depolarized/Live cells	0.0001	0.1604	0.0008	0.8802	0.0015	0.0753
Depolarized/Dead cells	0.0089	0.0340	0.0001	0.9251	0.4512	0.0973
Total depolarized cells	0.0002	0.0187	0.0033	0.5289	0.0024	0.0092
Live cells	0.0057	0.0018	0.7539	0.9205	0.3485	0.3088
Dead cells	0.0002	0.3976	0.0028	0.1679	0.0031	0.0737
MAI	0.4482	0.9536	0.3604	0.4823	0.3668	0.4545
Early Apoptotic	0.2200	0.0728	0.0025	0.0097	0.1035	0.3765
Late Apoptotic	0.6378	0.9850	0.0013	0.3087	0.6176	0.5466
Total Apoptotic	0.2970	0.1654	0.0017	0.0143	0.1319	0.4291

**Fig. 2:**
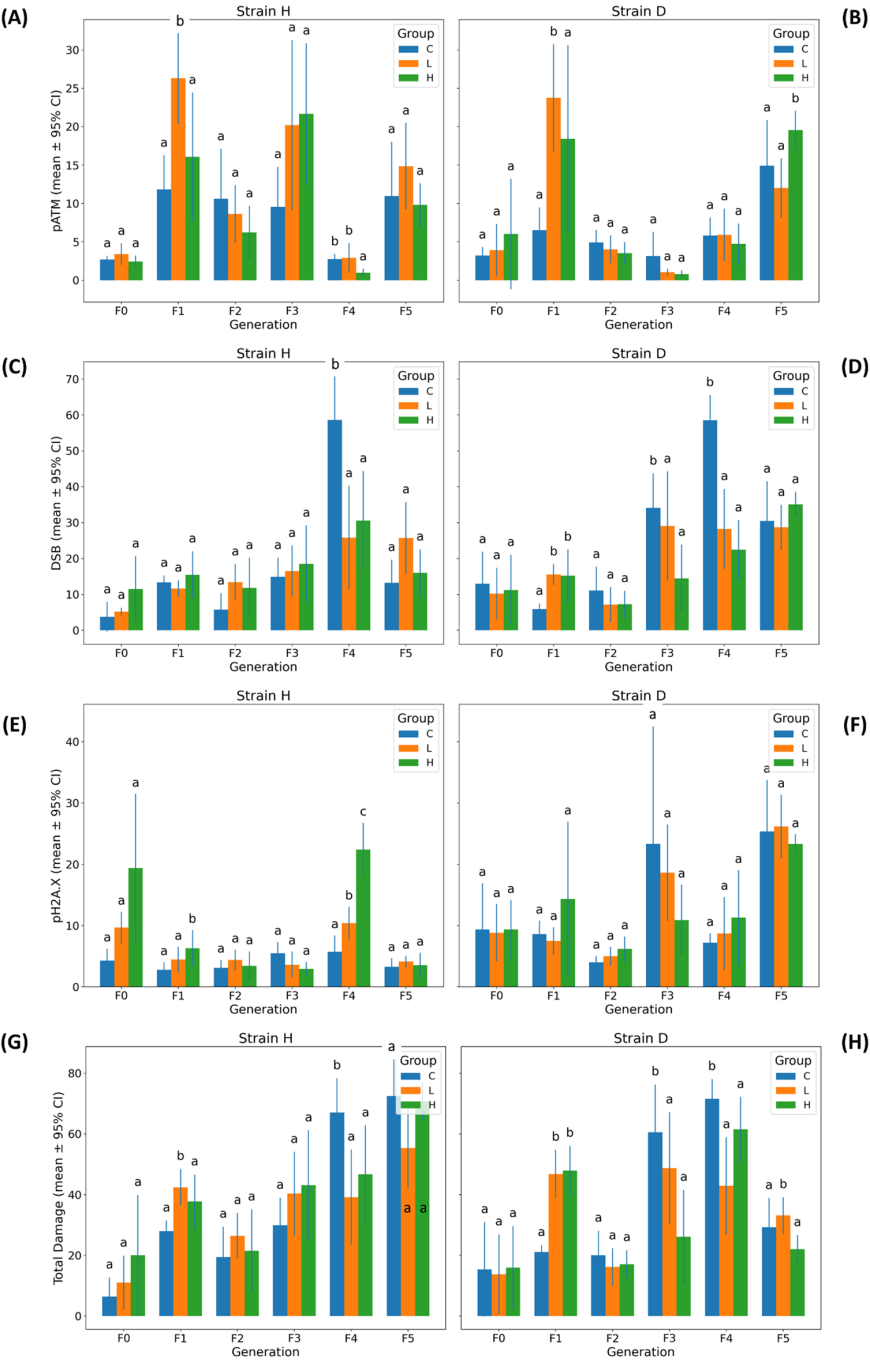
DNA damage biomarkers measured in gut cells of wild (**H**) and long-lived strain (**D**) of *Acheta domesticus*. Panels show mean ± 95% CI for four DNA-damage–related parameters: (**A**,** B**) pATM, (**C**,** D**) DSB, (**E**,** F**) pH2A.X, and (**G**,** H**) Total Damage, measured in strains H (left column) and D (right column) across generations (F0–F5) and treatment groups (**C**,** L**,** H**). Abbreviations: Generation F0-F4: (**C**) control animals fed uncontaminated food; (**L**) lower and (H) higher groups of animals fed GO-contaminated food at a concentration of 0.02 or 0.2 mg∙kg^–1^ of dry food, respectively; F5 – animals fed uncontaminated food. Values represent raw data (not normalized), and group differences within each strain × generation combination were assessed using permutation-based ANOVA (PERM-ANOVA; Freedman–Lane method, 9999 permutations). Letters above bars denote results of post-hoc pairwise permutation tests with Benjamini-Hochberg FDR correction; groups that do not share a letter differ significantly (*p* < 0.05). These analyses correspond to the univariate PERM-ANOVA results described in the Statistical Analysis section.

In both strains, in the F1 generation, the lower concentration resulted in a significant increase in pATM compared to the control. In the F3 generation, although no statistically significant differences were found, a distinct trend was evident in both strains – the H strain exhibited an increasing trend after treatment. In contrast, the D strain showed the opposite trend. Furthermore, in F5, a significant increase in pATM was observed in the H group of strain D, while strain H showed no significant differences between the GO-treated groups and the control (Fig. 2A, B).

Additionally, a multivariate repeated measures ANOVA revealed no significant global Strain × Group × Generation interaction; however, it did reveal significant Strain × Generation and Group × Generation interactions (Table S1). ANOVA analysis (Fisher test; *p* < 0.05) also showed the strongest response to GO in both strains in the F1 generation (Fig. S1), and significant differences between strains were observed in the F3 generation (Fig. S2).

#### Double-strand breaks (DSB)

DSB levels showed substantial temporal variability but no significant global Strain × Group × Generation interaction (three-way PERMANOVA: Pseudo-F = 1.596, pperm = 0.1135; Table 1). This indicates that, when all generations are considered simultaneously, strains H and D did not differ systematically in their multigenerational responses to GO exposure with respect to DSB formation. Generation-specific analyses, however, revealed several local but inconsistent interaction effects (Table 2). Two-way PERMANOVA was performed separately for each generation, showing marginal Strain × Group interactions in F1 (pperm = 0.0555) and F3 (pperm = 0.0845), whereas all other generations exhibited non-significant interactions (pperm > 0.10). These results suggest that strain-dependent responses in DSB occurred sporadically and did not follow a stable trend across generations.

In strain H, DSB levels tended to increase in generations F0–F3 in all experimental groups. The highest value was observed in the F4 control group, however, treatment with low or high GO concentrations significantly decreased DSB levels (Fig. 2C). In strain D, GO treatment at both concentrations significantly increased DSBs in the F1 generation, but, similarly to strain H, led to a significant reduction in DSBs in the F3 and F4 generations (Fig. 2D). Multivariate repeated measures ANOVA revealed no significant global Strain × Group × Generation interaction, confirming three-way PERMANOVA results. As with pATM, significant Strain × Generation and Group × Generation interactions were also found for DSBs (Table S1). ANOVA analysis (Fisher test; *p* < 0.05) confirmed a significant reduction in DSBs under the influence of GO in the F4 generation in both strains and in the F5 generation in strain H (Fig. S1). The expected marginal means analysis indicated significantly higher general DSB in the D strain compared to the H strain in F3 and F5 (Fig. S2B).

#### Histone H2A.X phosphorylation (pH2A.X)

pH2A.X levels showed considerable temporal variability but no significant global Strain × Group × Generation interaction (three-way PERMANOVA: Pseudo-F = 0.892, pperm = 0.5574; Table 1). This indicates that, when assessed across all generations simultaneously, strains H and D did not differ systematically in their multigenerational responses to GO exposure in terms of pH2A.X. Generation-specific two-way PERMANOVA revealed only a single significant Strain × Group interaction, occurring in F4 (pperm = 0.0381), while all other generations showed clearly non-significant effects (pperm > 0.11; Table 2). These results demonstrate that strain-dependent differences in pH2A.X occurred only locally and did not form a consistent generational pattern.

The level of pH2A.X was generally lower in strain H compared to strain D (Fig. 2E, F). This was confirmed by ANOVA analysis, as shown in the Expected Marginal Means for generations F1, F3, and F5 (Fig. S2). Treatment of insects from strain H with GO revealed a tendency to increase the level of this parameter in generations F0, F1, and F4, with significant differences revealed in F1 (group H vs. C) and F4 (groups L and H vs. C) (Fig. 2E). Classical analysis of variance proved to be less sensitive. It revealed a significantly higher level of pH2A.X only in group H compared to C and only in generations F0 and F4 (Fig. S1E, F). In strain D, Permanova did not detect a significant effect of GO on the pH2A.X level in any of the tested generations (Fig. 2F). At the same time, classical ANOVA indicated a significantly lower level in group H compared to C only in the F3 generation (Fig. S1F).

#### Total damage

Total Damage showed pronounced temporal variation and was the only DNA-damage parameter with a significant global Strain × Group × Generation interaction (three-way PERMANOVA: Pseudo-F = 2.470, pperm = 0.0083; Table 1). This indicates that, when all generations are assessed jointly, strains H and D differed systematically in their multigenerational responses to GO with respect to cumulative DNA damage. Generation-specific two-way PERMANOVA confirmed this pattern, revealing significant Strain × Group interactions in F3 (pperm = 0.0183) and F5 (pperm = 0.0243), with an additional near-significant effect in F1 (pperm = 0.0522; Table 2). Multivariate repeated measures ANOVA also confirmed the significance of these interactions (Table S1).

Total Damage in the H strain in the control groups increased across generations, with GO causing a significant increase in this parameter only in the F1 generation after exposure to the lower concentration. In the remaining generations, GO caused no significant changes or reduced the value of this parameter. Significantly lower levels of Total Damage in this strain occurred in groups L and H compared to the control, only in the F4 generation (significance of differences confirmed by both PERM-ANOVA and ANOVA; Fig. 2 and Fig. S1).

Fluctuations in Total Damage in the control groups between generations were also observed in line D. However, in the F1 generation, both GO concentrations resulted in a significant increase in Total Damage. Moreover, in the F3 and F4 generations, GO caused a significant decrease in this parameter compared to the control (Fig. 2 and Fig. S1).

### Mitochondrial transmembrane potential

#### Cell viability

Live Cells exhibited a significant global Strain × Group × Generation interaction (three-way PERMANOVA: Pseudo-F = 1.987, pperm = 0.036; Table 1 and Table S2), indicating that the two strains differed in their overall multigenerational viability response to GO. Dead Cells showed an even more substantial and highly significant global interaction (Pseudo-F = 6.376, pperm = 0.0001; Table 1 and Table S2), demonstrating substantial and consistent strain-dependent differences in this parameter. Generation-specific analyses supported these findings (Table 2). For Live Cells, significant Strain × Group interactions were observed only in the early stages of exposure, occurring in F0 (pperm = 0.0057) and F1 (pperm = 0.0018), with no significant effects in later generations (pperm > 0.30). In contrast, Dead Cells showed recurrent significant interactions across multiple generations, including F0 (pperm = 0.0002), F2 (pperm = 0.0028), and F4 (pperm = 0.0031), with additional near-significant differences in F3 and F5.

**Fig. 3:**
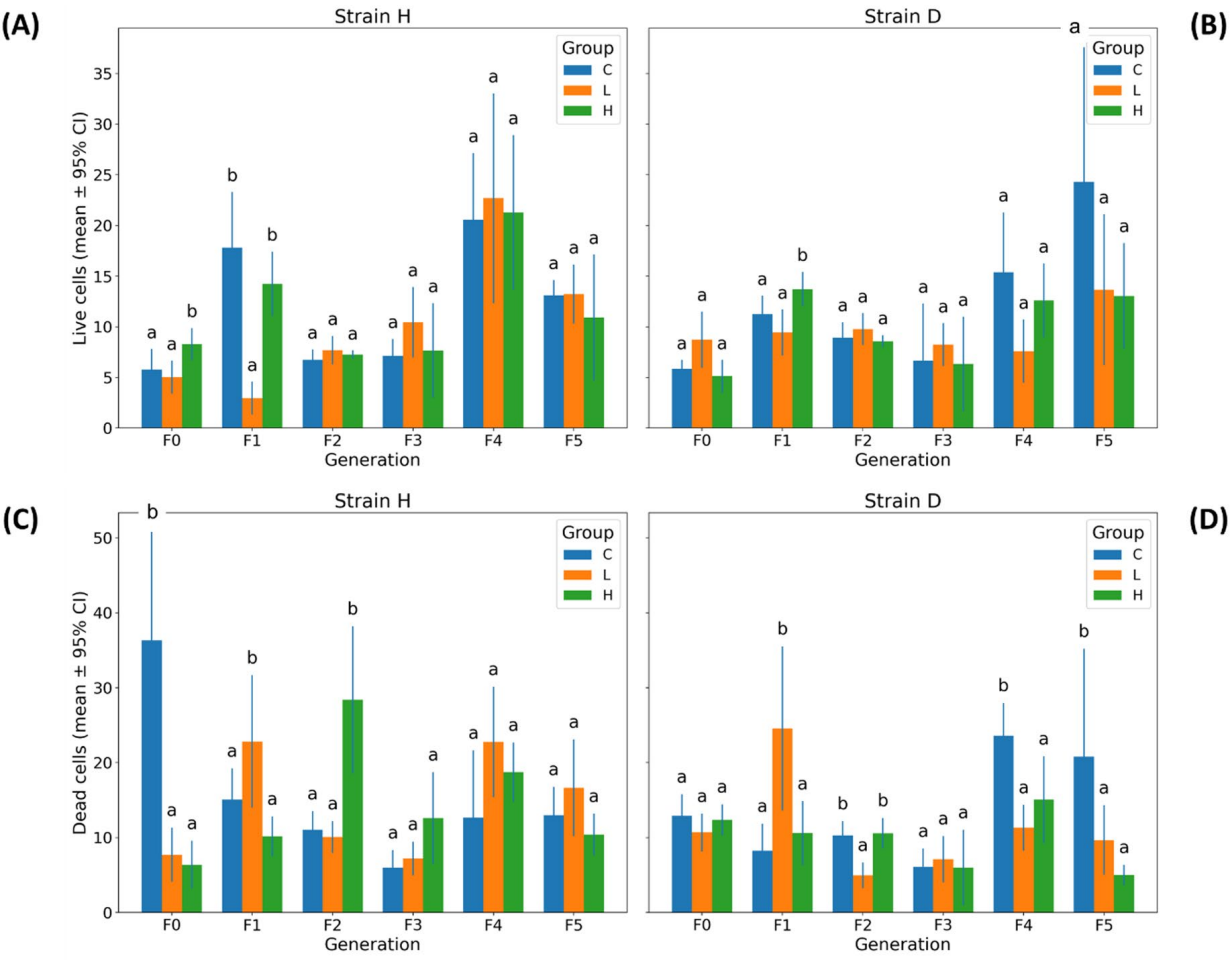
Live and dead gut cells in wild (H) and long-lived (D) strains of *Acheta domesticus* across generations. Panels depict mean ± 95% CI for (**A**,** B**) Live Cells and (**C**,** D**) Dead Cells measured in strains H (left column) and D (right column) across generations (F0–F5) and treatment groups (**C**,** L**,** H**). Abbreviations: see Fig. 2.

The abundance of live and dead cells in the control groups of both strains exhibited intergenerational fluctuations (Fig. 3), a typical phenomenon frequently observed across many parameters. Significant differences in live cell counts in response to GO exposure appeared only in isolated generations - namely in F0 (H strain, significantly higher live cell counts in group H compared with C) or in F1 (both strains; H strain, significantly fewer live cells in group L relative to C; D strain, significantly more live cells in group H compared with C; Fig. 3). Visualization of relative values revealed a GO effect only in generation F1 in strain H, as well as a tendency toward reduced live cell abundance in generations F4 and F5 in strain D (statistically supported only by the parametric ANOVA test; Fig. S3).

The proportion of dead cells in strain H was highest in generation F0 (control group), and GO supplementation led to a strong and significant reduction of this parameter in that generation (Fig. 3C). In subsequent generations, GO either increased dead cell abundance (F1 – group L vs. C; F2 – group H vs. C) or exerted no significant effect (generations F3–F5). In strain D, the lower GO concentration increased dead cell counts in F1, followed by a decrease in F2 compared to the control. Later, in generations F4 and F5, both GO concentrations resulted in a significant reduction in the proportion of dead cells (Fig. 3 and Fig. S3).

#### Depolarized live and depolarized dead cells

All three depolarization-related parameters showed substantial and highly significant global Strain × Group × Generation interactions, as demonstrated by the three-way PERMANOVA results (Depolarized/Live Cells: Pseudo-F = 5.786, pperm = 0.0001; Depolarized/Dead Cells: Pseudo-F = 5.890, pperm = 0.0001; Total Depolarized Cells: Pseudo-F = 7.204, pperm = 0.0001; Table 1 and Table S2). This indicates that strains H and D differed markedly in their multigenerational responses to GO exposure in terms of mitochondrial depolarization, and that these differences were robust across all measured indices.

Generation-specific two-way PERMANOVA further supported this pattern, revealing frequent and recurrent significant Strain × Group interactions across multiple generations. For Depolarized/Live Cells, significant interactions occurred in F0 (pperm = 0.0001), F2 (pperm = 0.0008), and F4 (pperm = 0.0015), with an additional near-significant effect in F5 (pperm = 0.0753). For Depolarized/Dead Cells, significant effects were observed in F0 (pperm = 0.0089), F1 (pperm = 0.0340), and F2 (pperm = 0.0001), while later generations showed weaker or non-significant interactions. Total Depolarized Cells exhibited the strongest pattern, with significant strain-dependent treatment responses in F0 (pperm = 0.0002), F1 (pperm = 0.0187), F2 (pperm = 0.0033), and F4 (pperm = 0.0024), as well as a near-significant effect in F5 (pperm = 0.0092) (Table 2).

**Fig. 4:**
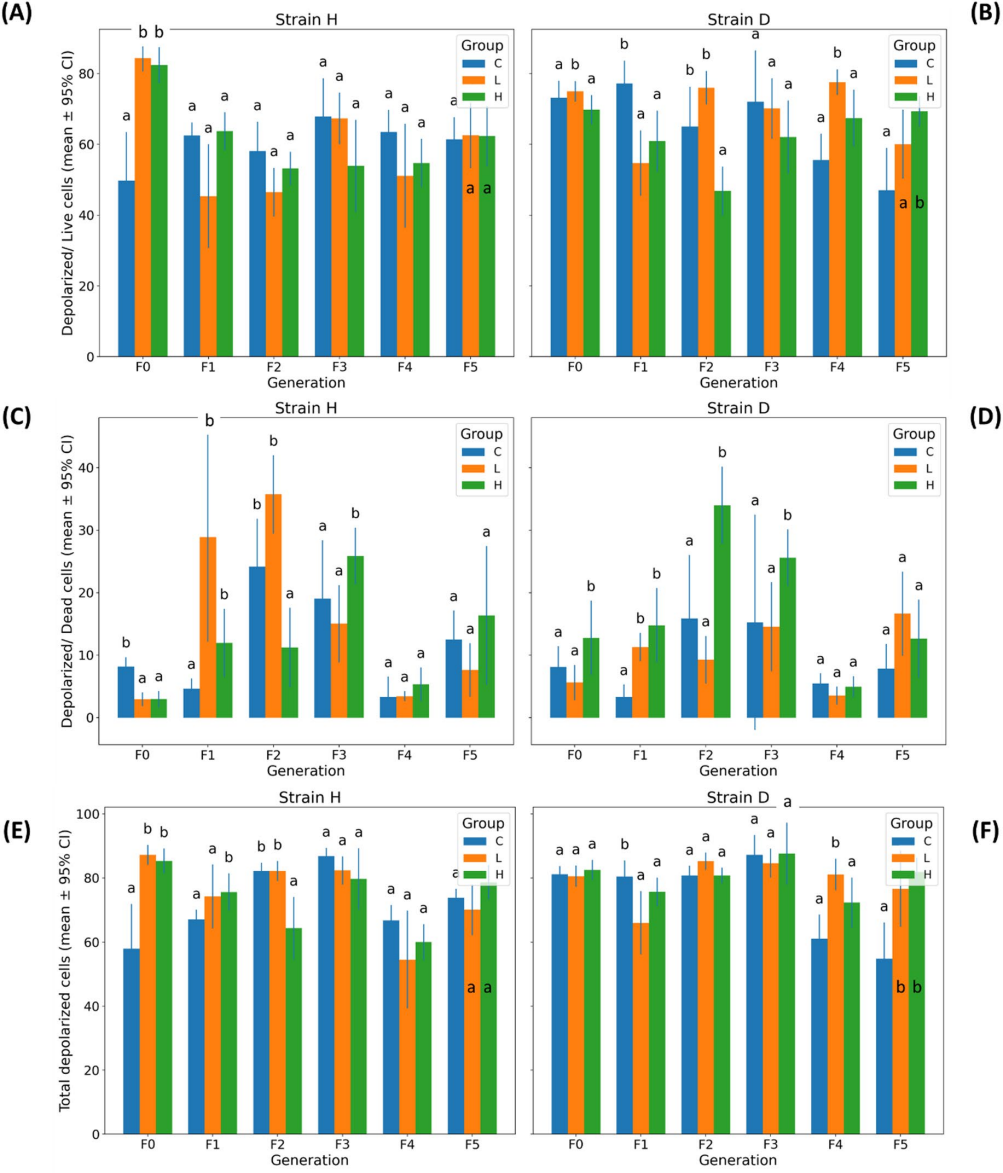
Mitochondrial membrane depolarization in gut cells of wild (H) and long-lived (D) strains of *Acheta domesticus* across generations. Panels show mean ± 95% CI for three depolarization-related parameters: (**A**,** B**) Depolarized/Live Cells, (**C**,** D**) Depolarized/Dead Cells, and (**E**,** F**) Total Depolarized Cells, measured in strains H (left column) and D (right column) across generations (F0–F5) and treatment groups (C, L, H). Abbreviations: see Fig. 2.

In strain H, GO induced a significant increase (confirmed by PERMANOVA) in the proportion of Depolarized/Live Cells only in generation F0 (Fig. 4A). In subsequent generations, this parameter remained comparable to the control, with a tendency toward decreased values in the treated groups (F1–F4; Fig. S4A). In contrast to strain H, strain D showed an opposite pattern: after a marked reduction in Depolarized/Live Cells in generations F0–F2 (particularly under the higher GO concentration), an increase was observed in generations F4 or F5 in either the L or H groups (Fig. 4B and Fig. S4B).

The most pronounced effect was the increase in Depolarized/Dead Cells in both strains in response to the lower and/or higher GO concentration in generations F1–F3. In later generations, GO exposure (F4) or its withdrawal (F5) did not induce significant changes in this parameter (Fig. 4C and D, and Fig. S4C and D).

In strain H, Total Depolarized Cells increased under GO exposure in F0 and F1. In F2, the higher dose reduced this parameter, and in subsequent generations, GO did not elicit significant changes (Fig. 4E and Fig. S4E). Notably, an opposite response was observed in strain D, where in generations F4, GO supplementation (at the lower and/or higher concentration) or its withdrawal (F5) led to an increase in Total Depolarized Cells (Fig. 4F).

Significant inter-strain differences across all experimental groups, assessed within a single analysis, occurred in generations F4 (all parameters) and F5 (Depolarized/Live Cells), as well as in F2 (Depolarized/Live Cells) (Fig. S5A-C).

### Mean autophagy intensity

Autophagy, assessed via the MAI index, showed no significant global Strain × Group × Generation interaction (three-way PERMANOVA: Pseudo-F = 0.720, pperm = 0.5964; Table 1 and Table S3), indicating the absence of systematic multigenerational differences between strains in their autophagic responses to GO exposure. Generation-specific two-way PERMANOVA results further supported this pattern, with all Strain × Group interaction terms remaining non-significant across generations (F0–F5: pperm > 0.36; Table 2).

**Fig. 5:**
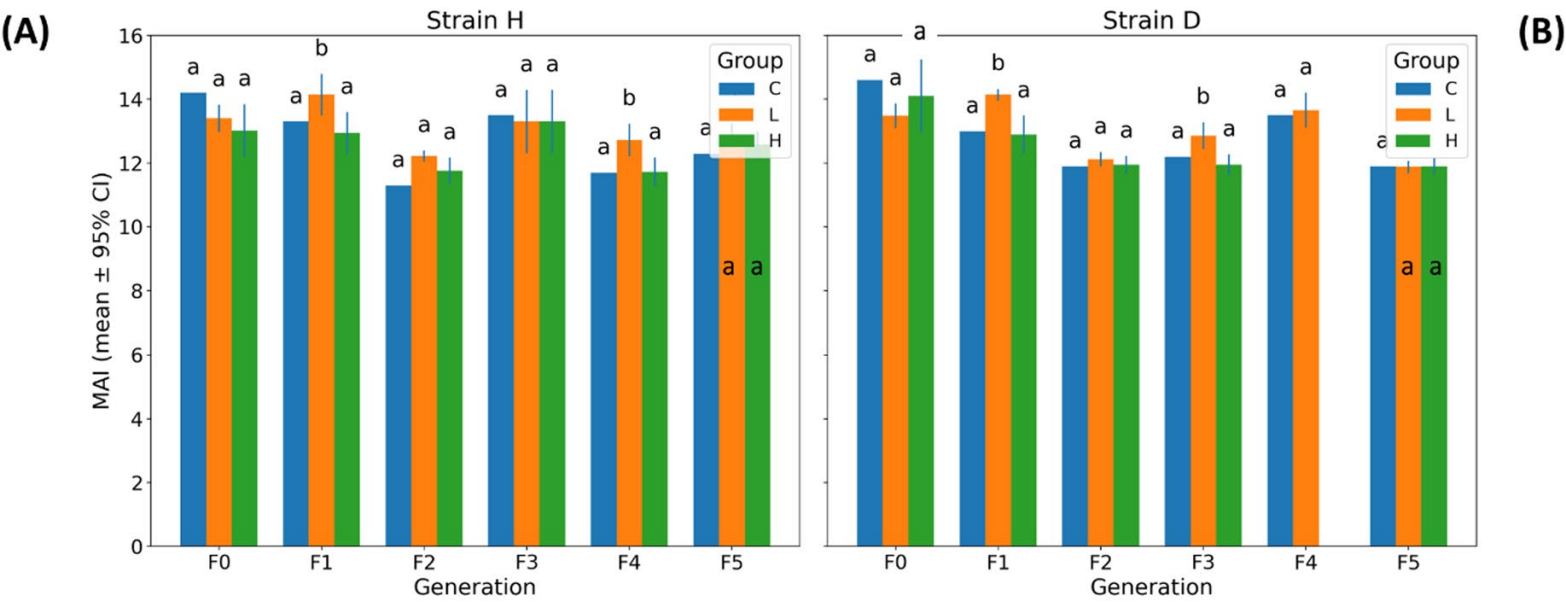
Autophagy activity (MAI index) in gut cells of wild (H) and long-lived (D) strains of *Acheta domesticus* across generations. Panels show mean ± 95% CI for the Mitophagy/Autophagy Index (MAI) measured in strains H (left) and D (right) across generations (F0–F5) and treatment groups (C, L, H). Abbreviations: see Fig. 2.

Overall, MAI levels were comparable between the two strains across all generations and experimental groups (Fig. 5 and Fig. S6). Pairwise permutation tests performed separately for each strain and generation revealed a significant effect of the lower GO concentration compared with the control in generations F1 and F4 (strain H), as well as in generations F1 and F3 (strain D). Comparison of expected marginal means demonstrated strain-specific differences in generations F3 and F5, with higher MAI values observed in strain H (Fig. S7).

### Health status of gut cells: apoptosis

#### Early apoptotic cells

Early apoptotic cell fractions showed moderate generational variability with significant global Strain × Group × Generation interaction (three-way PERMANOVA: Pseudo-F = 2.264, pperm = 0.0218; Table 1 and Table S4). Generation-specific two-way PERMANOVA analyses (Table 2) revealed that significant Strain × Group interactions occurred only in F2 (pperm = 0.0025) and F3 (pperm = 0.0097), with no evidence of interaction in the remaining generations (pperm > 0.07).

**Fig. 6:**
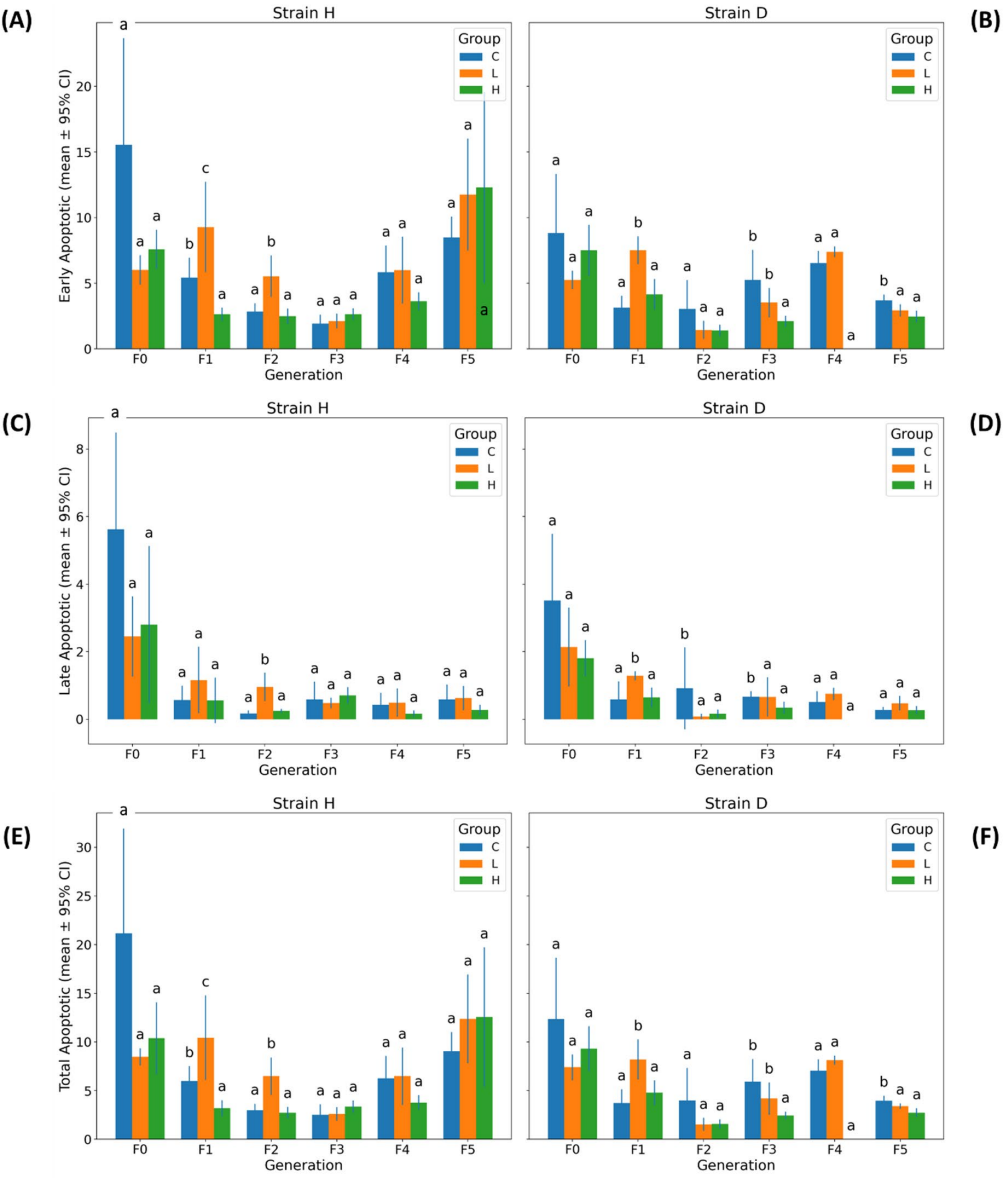
Apoptotic cell fractions in gut cells of wild (H) and long-lived (D) strains of Acheta domesticus across generations. Panels show mean ± 95% CI for three apoptosis-related parameters: (**A**,** B**) Early Apoptotic Cells, (**C**,** D**) Late Apoptotic Cells, and (**E**,** F**) Total Apoptotic Cells, measured in strains H (left column) and D (right column) across generations (F0–F5) and treatment groups (C, L, H). Abbreviations: see Fig. 2.

In strain H, the lower GO concentration caused a significant increase in the proportion of early apoptotic cells, but only in generations F1 and F2. In contrast, the higher concentration led to a significant decrease in this parameter exclusively in F1 (Fig. 6A).

Similarly, in strain D, the lower concentration induced a significant increase in early apoptotic cells in F1 (Fig. 6B). However, in subsequent generations, the pattern of GO-induced responses in strain D differed somewhat from that observed in strain H (Fig. 6A, B, and Fig. S8A, B). Beginning in F2, a tendency toward reduced early apoptotic cell fractions emerged, although statistically significant differences were detected only in F3 (H vs. C) and in F5 (L and H vs. C).

Comparison of Expected Marginal Means between strains (Fig. S9A) showed significantly higher levels of early apoptotic cells in generations F0 and F5.

#### Late apoptotic cells

Late apoptotic cell fractions showed variation across generations but no significant global Strain × Group × Generation interaction (three-way PERMANOVA: Pseudo-F = 0.827, pperm = 0.5983; Table 1; Table S4). Generation-specific two-way PERMANOVA revealed a single significant Strain × Group interaction occurring in F2 (pperm = 0.0013), with all remaining generations showing non-significant effects (pperm > 0.30; Table 2).

The effects of GO on the proportion of late apoptotic cells were sporadic (Fig. 6C, D and Fig. S8C, D). In strain H, significant differences were observed only in F2, where the lower GO concentration resulted in a higher mean value compared to the control. In strain D, the lower dose significantly increased this parameter in F1, whereas in F3 and F4, GO exposure led to a reduction in late apoptotic cell fractions.

#### Total apoptotic cells

Total apoptotic cell fractions exhibited a statistically significant global Strain × Group × Generation interaction (three-way PERMANOVA: Pseudo-F = 1.995, pperm = 0.0453; Table 1; Table S4). Generation-specific two-way PERMANOVA analyses revealed significant Strain × Group interactions in two generations only: F2 (pperm = 0.0017) and F3 (pperm = 0.0143), whereas all remaining generations showed non-significant differences (pperm > 0.13; Table 2).

For both strains, H and D, the overall patterns of total apoptotic cell proportions closely resembled those observed for early apoptotic cells in the GO-treated groups (Fig. 6A-B, E-F, and Fig. S8A-B, E-F).

Comparison of Expected Marginal Means between strains within each generation revealed significantly lower total apoptotic cell levels in strain D compared with strain H in generations F0 and F5 - mirroring the pattern previously noted for early apoptotic cells (Fig. S9B).

### Multivariate response patterns

**Fig. 7:**
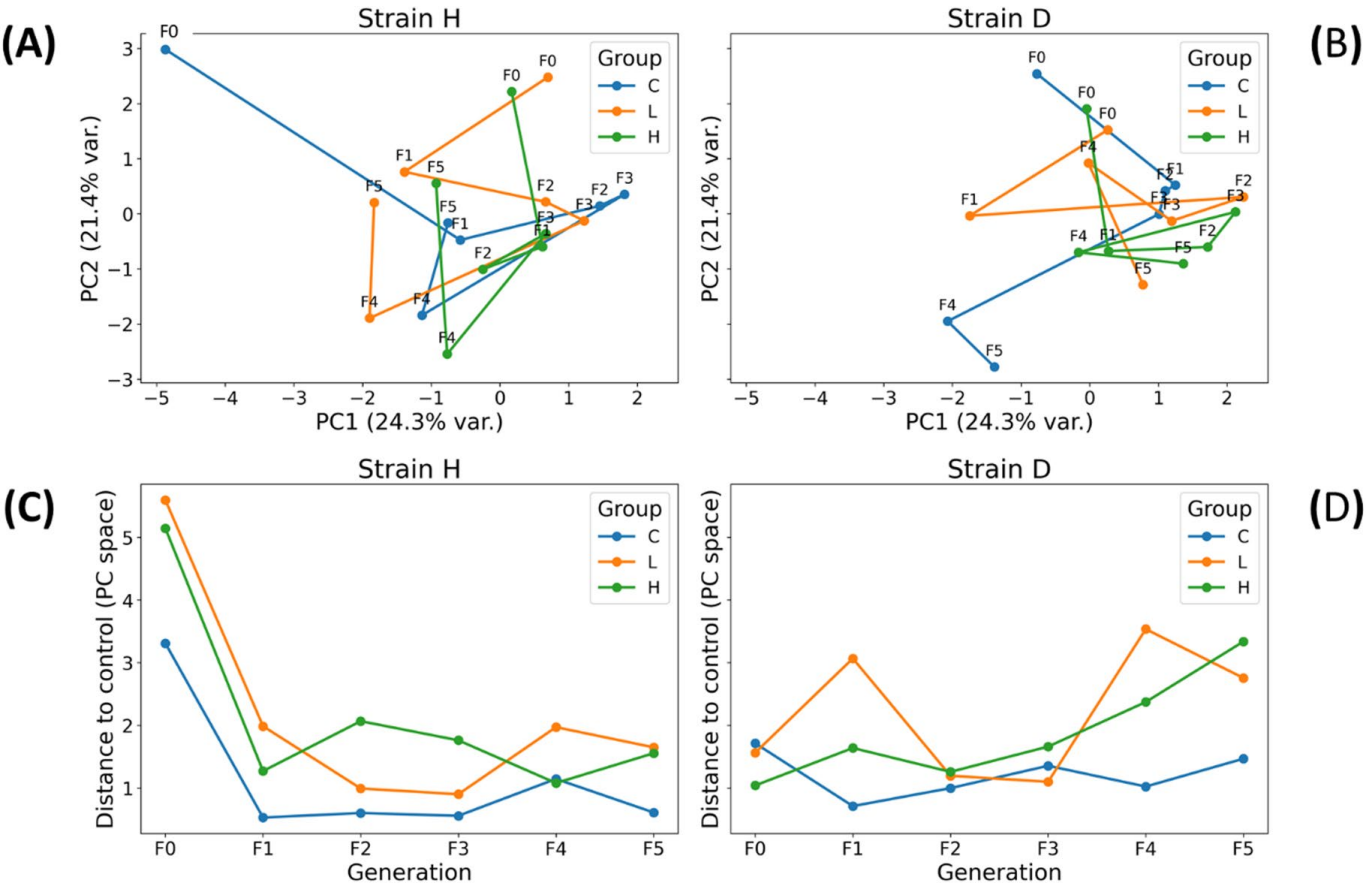
Multivariate response patterns of cellular traits in wild (H) and long-lived (D) strains of *Acheta domesticus* across generations. (Top panels) Principal Component Analysis (PCA) plots based on all 13 measured variables show trajectories of multigenerational shifts in the multivariate phenotype for each treatment group (C, L, H). Points represent generation-specific centroids, connected chronologically (F0→F5). PCA was performed on standardized variables, capturing 24.3% (PC1) and 21.4% (PC2) of total variance. (Bottom panels) Distance-to-control plots illustrate the Euclidean distance in PCA space between treated groups (L, H) and the control (C) within each generation, providing a generation-by-generation measure of multivariate deviation from baseline. Abbreviations: See Fig. 2. All multivariate tests were performed using PERMANOVA (Freedman-Lane method, 9999 permutations) with Euclidean distances.

The PCA trajectories (Fig. 7A-B) illustrate multigenerational shifts in the combined cellular parameters of *A. domesticus* across generations F0 to F5. In both strains, F0 – the first generation exposed to GO – was positioned far from subsequent generations in PCA space, indicating a strong initial multivariate perturbation. In strain H, the control group in F0 was markedly separated from the GO-exposed groups (L and H), demonstrating a pronounced effect of GO in the first generation. This degree of separation among experimental groups was not observed in strain D.

In strain H, the trajectory showed a pronounced shift from F0 to F1, followed by progressive convergence of generations F2–F4, suggesting partial stabilization or acclimation of the multivariate phenotype under continued exposure. A distinct cluster is visible in F4, comprising closely grouped C, L, and H groups (Fig. 7A). Notably, F5 – the recovery generation fed uncontaminated food – shifted away from the F4 cluster, indicating a distinct multivariate response associated with GO withdrawal.

In strain D, intergenerational transitions were more compact, with smaller distances between consecutive generations compared with strain H. Although F0 was again positioned separately from later generations, generations F1-F4 formed a relatively tight cluster, suggesting a more moderate or buffered multivariate response during prolonged GO exposure. Similar to strain H, the F5 generation deviated from the F4 generation, reflecting phenotypic restructuring following the cessation of GO supplementation (Fig. 7B).

The distance-to-control plots (Fig. 7C-D) quantify the magnitude of multivariate deviation from the control insects within each generation. In strain H, both GO concentrations caused a pronounced increase in distance in F0, followed by a decline in F1 and relatively small fluctuations across F2–F5. This pattern indicates a strong initial perturbation, with partial stabilization under ongoing exposure and a moderate shift in the recovery generation (F5).

In strain D, multivariate distances were generally lower and exhibited more minor generational fluctuations than in strain H, suggesting a more buffered response to GO exposure. A mild increase in distance was observed in F4-F5 (stronger for the higher dose in F5), indicating delayed divergence or a recovery-associated shift.

## Discussion

### GO treatment effect

GO intoxication in two concentrations for five generations significantly affected most of the investigated parameters of crickets’ health status. The results support the first hypothesis’s acceptance: “*DNA damage and/or cell viability and/or mitopotential and/or autophagy and/or apoptosis gut health parameters are impacted by GO applied in two concentrations*,* and the effects may vary between the generations.*”

The main effects analysis for all investigated parameters revealed that the interaction between the factor group and generation had a significant effect. This result clearly shows that the GO impacts insect health, and the effects vary across generations (Table S1-S4). Additionally, the PCA analysis revealed that the clusters of the GO groups are shifted away from the control and differ across generations (Fig. 7C, D). The dependence of centroid distances allows us to distinguish three types of responses among the studied generations (Fig. 7A–B). The first type of response is associated with the initial contact with the toxicant (F0). The next kind of response is related to long-term exposure of individuals to GO (F1–F3) and the attempt to establish a new equilibrium (F4). It is worth emphasizing that the digestive tract is the organ most exposed to the effects of GO, as it is the site of direct contact. GO may act only on the intestine; however, it cannot be excluded that, together with the hemolymph that bathes the organs, it may reach, for example, the gonads and the eggs. The effects of such transfer may result in egg necrosis, changes in the proportion of egg nutrients, lower hatching rates, or even lower body mass in the next generation^24,25^. However, in the studies cited, the GO dose was high. Although the low GO concentrations we used do not rule out the possibility of transferring to other organs, it can be assumed that it is less likely. A different response of the next generation to GO may be mediated through epigenetic changes. The F0 was first exposed to the stressor. It was forced to confront the burdensome effects that led to DNA damage (Fig. 2). Graphene oxide can induce DNA damage, such as double-strand breaks (DSBs), either indirectly through the production of reactive oxygen species (ROS) or directly by binding to the DNA structure and causing cuts with its sharp edges^26^. Additionally, the imbalance in fighting against the stressor was evident in mitochondrial potential changes in F0, presenting a high level of depolarization in live cells and total depolarization (Fig. 4A, C). Mitochondria are particularly vulnerable to oxidative stress caused by GO, which can disrupt the function of the respiratory chain and mitochondrial membrane potential. In the work of Szmidt et al^27^. using the chicken embryo model, GO treatment caused mitochondrial leakages and intramitochondrial inclusions. Mitochondrial potential dysfunction was also observed in our previous work, in insects at various developmental stages (larvae, young adults, and mature adults)^28^. In the situation of imbalance, with activated paths of pH2A.X due to DNA DSB, and mitochondrial potential disruption, autophagy or apoptosis could be activated^29^. As a result, the viability of the cells could drop. In the presented studies, the autophagy level in the F0 group remained unchanged (Fig. 5), while the proportion of apoptotic cells (Fig. 6) and dead cells (Fig. 3) decreased significantly. A likely explanation for this phenomenon may not involve the “inhibition” of apoptosis per se, but rather its “postponement in time.” ATM kinase mobilizes and regulates the cellular response to DSBs by activating cell cycle checkpoints^30^. In this case, the time window was created to allow for the DNA repair process. If the process failed, the apoptosis was activated.

The GO concentrations used in the experiment were low but high enough to cause changes in mitopotential (Fig. 4). They had no impact on the activation of apoptosis or autophagy (Figs. 5 and 6) in F0 cells. This may suggest that, in F0, despite the presence of a stressor, the organism can actively strive to maintain homeostasis and is involved in repair processes, which delay the activation of apoptosis over time.

The situation appears to change significantly in the response of the next generations, F1, F2, and F3. The picture of these generations shows that the struggle to maintain homeostasis is only beginning at this stage. The imbalance is maintained, and it is presented not only as higher DNA damage (Fig. 2) but also as a higher proportion of depolarized dead cells (Fig. 4C, D) and a high level of total apoptotic cells (Fig. 6E, F). The apoptosis was activated, and the viability of cells decreased significantly (Fig. 3). The picture of the struggle against prolonged exposure to GO in the diet showed that in the F0, the concentration used is not that important – the protection and repair systems turned on regardless of the GO concentration level, and the answer was similar. It appears that in F1 to F3, the imbalance caused by the stressor remains challenging to eliminate. Moreover, the lower and higher GO concentrations caused varied effects on measured parameters (Figs. 2, 3, 4, 5 and 6). Particular attention should be paid to the lower GO concentration, which causes more pronounced effects of intoxication. At low GO concentrations, cells may not recognize the stress factor properly, which is not strong enough to trigger repair, detoxification, or adaptive mechanisms. In that situation, lower GO concentration caused more substantial adverse effects in the organism compared to the groups treated with higher GO concentrations. In our previous work, the multigenerational GO effects were investigated in the gut enzymes of the house cricket^31^. In the study, a lower dose of GO elicited a greater stimulatory effect on the activity of gastrointestinal enzymes than a higher dose. It supports the notion that GO does not always exert its impact in a conventionally expected dose-dependent manner, and low GO doses should be considered as not neutral to the organism. An explanation for this phenomenon may also be related to GO aggregation at higher concentrations, which in turn may hinder its ability to penetrate the cell membrane^31^.

In the PCA analysis, the centroids of the F4 generation are located within a shared cluster, which indicates a modified response (particularly in the wild-type strain) (Fig. 7A). It may reflect the establishment of a new homeostatic equilibrium. Most of the investigated cell health parameters were similar to those of the controls or even presented lower values (Figs. 2, 3, 4, 5 and 6).

Under prolonged stress conditions, selection may occur - and most likely does - favoring individuals that cope more effectively with the stressor, particularly in organisms exhibiting an r-selected life-history strategy, as is the case for insects, including *A. domesticus*. This species naturally displays high developmental mortality^22^. In our experiment, a slight increase in mortality was observed in generations F2 and F3, but only in the H strain (unpublished data). Nevertheless, despite this selective pressure, our focus remains on the individuals that survived and on the mechanisms that enabled their survival. At this stage, it remains an open question which mechanisms responsible for coping with stress were retained across generations. At the F4 (presenting the new homeostatic equilibrium), the stressor was still present, but the organism could fight and protect itself. One possible explanation for this phenomenon is epigenetic modifications. Post-transcriptional epigenetic modifications are a crucial mechanism for regulating tissue-specific gene expression. It is well-documented that environmental influences, such as diet or exposure to toxic pollutants, can alter the epigenome. That can allow the environment to influence gene expression and cell function. Exposure-acquired epigenetic patterns can persist and be inherited by subsequent generations^14^. Intergenerational transmission occurs over two generations, where the impacts of an exposure in the parent are directly inherited by the offspring^32^. We believe the multigenerational administration of food with GO changed epigenetic patterns passed to the offspring, and a new homeostatic equilibrium has been established. Our previous work investigated the effects of GO multigenerational intoxication on DNA stability and global DNA methylation^17^. The parameters of DNA damage showed increased AP sites and 8-OHdG lesions in the first three generations, and lower in the next ones. However, the pattern of DNA methylation was comparable across every generation, suggesting that other epigenetic mechanisms may be involved^17^. The lack or presence of global methylation changes may be related to exposure time, experimental models, nanoparticle types, and sizes^33^.

In the context of DNA methylation, increasing attention is being given to gene-specific methylation changes, as well as to alterations in the expression of DNA methyltransferases and TET enzymes^34–37^. The potential epigenetic effects of GO are still poorly understood. Still, some mechanisms have been proposed: DNA methylation, changes in methylation enzyme activity, histone modifications including H4 acetylation and H1 phosphorylation, changes in profiles of miRNA and lncRNA, and others^38^.

The model of epigenetic changes as a factor increasing the chances of survival and persistence under unfavorable conditions is highly plausible when considering the modification of the response to a stressor^39,40^. Additionally, mutations and recombination cannot be ruled out; however, if they are beneficial, their expression requires more time than just a few generations. Still, it is essential to acknowledge the role of selection, whereby only the less sensitive individuals can complete the full life cycle and reproduce - a key process shaping populations in changing environments^41^. However, the present study was designed to investigate the molecular mechanisms underlying the multigenerational response to GO exposure, and the process of natural selection itself is not being questioned.

### Recovery generation effect

The main effects analysis revealed generation as a significant factor, with *p* < 0.001 (Table S1-S4). This confirms that the responses of all generations to the GO differed significantly. One of the generations was exceptional – the recovery generation (F5), where the stressor was removed, and insects were fed the control food. The phenomenon of a stressor’s “withdrawal” from the natural environment is possible. Various substances considered to be toxic materials can undergo degradation with the involvement of microorganisms. The same may also occur with GO^42,43^. The obtained results showed that in F5, for most experimental groups, there was no significant improvement in cell health status. Therefore, we rejected the hypothesis: *“The cessation of GO from the food in generation F5 is expected to restore the baseline levels of the examined health parameters.”* Following the completion of treatment, the organism is expected to return to baseline homeostasis; however, this does not always occur. In both strains, the GO groups were shifted from the controls (Fig. 7C, D). It appears that the cessation of GO from food may act as a new stressor. The equilibrium that had developed over multiple generations was once again disrupted. Changes in cellular parameters may occur as a consequence of this phenomenon. In our previous work, one of the notable changes was DNA damage in the recovery generation, as measured by higher AP sites and 8-OHdG lesions in both lower and higher GO groups. Moreover, while global DNA methylation changes did not reach statistical significance, a clear trend toward decreased global DNA methylation was observed compared to the control^17^. In the work of Klibaner-Schiff et al.^14^, the effects of multigenerational exposure of *Caenorhabditis elegans* to silver nanoparticles showed that pronounced sensitization occurred in the next generations to the stressor. It is worth noticing that during studies of ten generations, starting from G6, the recovery generations were not treated with Ag. The necessary information was that the sensitivity to Ag resulting from the initial multigenerational exposure persisted in the recovery populations. Their response sensitivity for all endpoints was closely related to the last ancestral generation exposed. The authors suggested that the mechanisms of transgenerational sensitivity transfer are likely organized through the epigenome, including DNA methylation, histone modification, and miRNA expression, which can be affected by nanoparticle exposure^14^.

It is worth noting that the last generation was not exposed to GO in the diet; however, its reproductive stem cells were exposed. Such exposure may have observable consequences for this generation and for subsequent, already unexposed generations. Additionally, a deteriorated physiological condition in the mother may affect egg quality and, consequently, the condition of the offspring. Nevertheless, this remains an indirect effect of GO exposure, and any potential consequences for subsequent generations should be the subject of future studies.

### Strain effect

The effects of GO intoxication over five generations varied across GO concentrations and generations, as well as for the two investigated strains, wild and long-lived (Figs. 2, 3, 4, 5, 6 and 7). The PCA analysis revealed that strains react differently across generations (Fig. 7). Additionally, the expected marginal means analysis indicated statistically significant differences between strains (Fig. S2, S5, S7, S9). Our results support the hypothesis: *“Different life histories resulting from selection for longevity may influence the intensity of selected cellular responses to GO.”* Organisms selected for lifespan may differ in their response to stressors like GO due to variations in life-history strategies, energy allocation, defense mechanisms, and the activity of oxidative stress and DNA repair pathways. Long-lived stains develop more efficient repair and detoxification systems, potentially making them less sensitive to the effects of toxic nanoparticles. In contrast, wild-lived strains may respond more rapidly but have reduced capacity to cope with cellular damage, leading to increased sensitivity^9,44^.

In most investigated parameters, generations presented fluctuations in both strains (Figs. 2, 3, 4, 5 and 6). The long-lived strain is presumed to have more efficient and sophisticated DNA damage repair pathways than the wild type. This hypothesis is supported by the total DNA parameter, indicating that the D strain tends to normalize more quickly in F2 (Fig. 2G, H), possibly reflecting a more robust and organized defensive response than the wild-type strain (Fig. 2). The DNA repair rates may be correlated with lifespan^45,46^. In the study of two strains of *Caenorhabditis elegans*, the findings showed that DNA repair capacity is associated with longevity in *C. elegans*. The stress-resistant mutants presented more effective gene-specific repair of UV-induced pyrimidine dimers than the wild type^47^. Most of the differences in cellular parameters between the strains are concentrated in generations F3-F5 (Figs. S2, S5, S7, S9). These generations have been subjected to GO exposure over an extended period. This strengthens the hypothesis that following generations may be influenced by epigenetic modifications that serve as heritable signals of stressor exposure.

## Conclusion

Even at low doses, graphene oxide intoxication induced multilevel cellular responses in five subsequent generations. The stressor influenced DNA stability, mitochondrial potential, apoptosis, and viability, revealing that GO is not neutral to living organisms. Across five generations, we identified different types of responses: to GO that in first stage (F0) were characterized by DNA damage and mitochondrial depolarization, but limited apoptosis, likely due to delayed activation of cell death pathways in favor of DNA repair; second stage (F1-F3) where imbalance intensified, apoptosis increased, and viability decreased, especially in the lower GO concentration, suggesting that mild stress may bypass repair system activation and lead to stronger adverse outcomes; and last stage (F4) where cell stress parameters gradually returned to control levels, indicating the establishment of a new homeostatic state. GO cessation in recovery F5 acted as a new stressor, disturbing the previously achieved equilibrium. Like other xenobiotics, graphene oxide is most likely responsible for changes that persist over two to three generations. We believe that epigenetic inheritance is a likely mechanism underlying the multigenerational adaptation observed in GO-exposed insects, and future research should aim to elucidate this phenomenon in more detail.

## Supplementary Information

Below is the link to the electronic supplementary material.

Supplementary Material 1

## Data Availability

Raw data are provided on the RepOD database (accession: https://doi.org/10.18150/F3YO29).
