# Supplementary material for: Response to multigenerational graphene oxide exposure in acheta domesticus strains selected for longevity
Source: Sci Rep. 2026 Jan 30;16:6687. doi: 10.1038/s41598-026-37623-7 (PMC12913934; doi:10.1038/s41598-026-37623-7)
Supplement: Supplementary file 1 — Supplementary Material 1 [file 41598_2026_37623_MOESM1_ESM.docx]

**Supplementary data**

**Table S1.** Multivariate repeated measures ANOVA for Strain [1], Group [2], Generation [3], and interaction of the factors [1] × [2]; [1] × [3]; [2] × [3]; [1] × [2] × [3] on DNA damage measured in five generations of *Acheta domesticus* intoxicated with GO and one recovery generation. Symbols description: F - F ratio; df1 - treatment and error degrees of freedom, respectively; p - p-value, n=5.

| Effect | pATM | | | DSB | | | pH2A.X | | | Total damage | | |
| --- | --- | --- | --- | --- | --- | --- | --- | --- | --- | --- | --- | --- |
|  | F | df | *p* | F | df | *p* | F | df | *p* | F | df | *p* |
| Strain [1] | 5.84 | 1 | <0.001 | 6.35 | 1 | <0.001 | 36.03 | 1 | <0.001 | 3.10 | 1 | 0.806 |
| Group [2] | 4.52 | 2 | <0.001 | 3.62 | 2 | <0.001 | 2.31 | 2 | 0.103 | 0.40 | 2 | 0.672 |
| Generation [3] | 24.82 | 5 | <0.001 | 39.21 | 5 | <0.001 | 8.50 | 5 | <0.001 | 31.67 | 5 | <0.001 |
| [1] x [2] | 1.38 | 2 | 0.254 | 1.91 | 2 | 0.152 | 2.96 | 2 | 0.055 | 1.79 | 2 | 0.171 |
| [1] x [3] | 11.32 | 5 | <0.001 | 3.60 | 5 | <0.001 | 16.21 | 5 | <0.001 | 14.29 | 5 | <0.001 |
| [2] x[3] | 3.36 | 10 | <0.001 | 7.80 | 10 | <0.001 | 2.54 | 10 | <0.001 | 4.90 | 10 | <0.001 |
| [1] x [2] x [3] | 1.51 | 10 | 0.141 | 1.60 | 10 | 0.114 | 0.89 | 10 | 0.543 | 2.78 | 10 | <0.001 |

**Table S2.** Multivariate repeated measures ANOVA for Strain [1], Group [2], Generation [3], and interaction of the factors [1] × [2]; [1] × [3]; [2] × [3]; [1] × [2] × [3] on Mitopotential measured in five generations of *Acheta domesticus* intoxicated with GO and one recovery generation. Symbols description: F - F ratio; df1 - treatment and error degrees of freedom, respectively; p - p-value, n=5.

| Effect | Depolarized/Live | | | Depolarized/Dead | | | Total depolarized | | | Live | | | Dead | | | |
| --- | --- | --- | --- | --- | --- | --- | --- | --- | --- | --- | --- | --- | --- | --- | --- | --- |
|  | F | df | p | F | df | p | F | df | p | F | df | p | F | Df | p |  |
| Strain [1] | 11.42 | 1 | <0.001 | 0.78 | 1 | 0.379 | 11.27 | 1 | <0.001 | 0.94 | 1 | 0.333 | 10.65 | 1 | <0.001 |  |
| Group [2] | 0.67 | 2 | <0.001 | 4.92 | 2 | <0.001 | 4.29 | 2 | <0.001 | 2.23 | 2 | 0.111 | 2.18 | 2 | 0.117 |  |
| Generation [3] | 8.50 | 5 | 0.512 | 25.09 | 5 | <0.001 | 20.51 | 5 | <0.001 | 18.83 | 5 | <0.001 | 7.63 | 5 | <0.001 |  |
| [1] x [2] | 2.72 | 2 | <0.001 | 8.31 | 2 | <0.001 | 1.14 | 2 | 0.322 | 0.50 | 2 | 0.603 | 0.52 | 2 | 0.593 |  |
| [1] x [3] | 2.93 | 5 | 0.069 | 1.60 | 5 | 0.161 | 2.60 | 5 | <0.001 | 6.40 | 5 | <0.001 | 1.17 | 5 | 0.325 |  |
| [2] x[3] | 6.47 | 10 | <0.001 | 3.07 | 10 | <0.001 | 4.92 | 10 | <0.001 | 2.40 | 10 | <0.001 | 8.43 | 10 | <0.001 |  |
| [1] x [2] x [3] | 5.79 | 10 | <0.001 | 5.89 | 10 | <0.001 | 7.20 | 10 | <0.001 | 1.99 | 10 | <0.001 | 6.38 | 10 | <0.001 |  |

**Table S3.** Multivariate repeated measures ANOVA for Strain [1], Group [2], Generation [3], and interaction of the factors [1] × [2]; [1] × [3]; [2] × [3]; [1] × [2] × [3] on autophagy measured in five generations of *Acheta domesticus* intoxicated with GO and one recovery generation. Symbols description: F - F ratio; df1 - treatment and error degrees of freedom, respectively; p - p-value, n=5. Dashes denote cases where the software returned no test statistic for the main effect due to a zero sum of squares, while all estimable interaction terms are reported exactly as provided by the model.

| Effect | Mean autophagy intensity | | |
| --- | --- | --- | --- |
|  | F | df | p |
| Strain [1] | ― | ― | ― |
| Group [2] | 1.95 | 1 | 0.165 |
| Generation [3] | 19.82 | 4 | <0.001 |
| [1] x [2] | 0.29 | 1 | 0.589 |
| [1] x [3] | 3.22 | 4 | <0.001 |
| [2] x[3] | 2.00 | 9 | <0.001 |
| [1] x [2] x [3] | 0.81 | 9 | ― |

**Table S4.** Multivariate repeated measures ANOVA for Strain [1], Group [2], Generation [3], and interaction of the factors [1] × [2]; [1] × [3]; [2] × [3]; [1] × [2] × [3] on apoptosis measured in five generations of *Acheta domesticus* intoxicated with GO and one recovery generation. Symbols description: F - F ratio; df1 - treatment and error degrees of freedom, respectively; p - p-value, n=5. Dashes denote cases where the software returned no test statistic for the main effect due to a zero sum of squares, while all estimable interaction terms are reported exactly as provided by the model.

| Effect | Early apoptotic | | | Late apoptotic | | | Total apoptotic | | |
| --- | --- | --- | --- | --- | --- | --- | --- | --- | --- |
|  | F | df | p | F | df | p | F | df | p |
| Strain [1] | ― | ― | ― | ― | ― | ― | ― | ― | ― |
| Group [2] | 0.08 | 1 | 0.776 | 1.78 | 1 | 0.184 | 0.48 | 1 | 0.491 |
| Generation [3] | 23.07 | 4 | <0.001 | 39.86 | 4 | <0.001 | 28.23 | 4 | <0.001 |
| [1] x [2] | 0.25 | 1 | 0.618 | 0.11 | 1 | 0.740 | 0.15 | 1 | 0.697 |
| [1] x [3] | 10.98 | 4 | <0.001 | 1.92 | 4 | 0.111 | 7.59 | 4 | <0.001 |
| [2] x[3] | 4.96 | 9 | <0.001 | 3.59 | 9 | <0.001 | 5.04 | 9 | <0.001 |
| [1] x [2] x [3] | 2.28 | 9 | <0.001 | 0.91 | 9 | 0.518 | 2.03 | 9 | <0.001 |

***
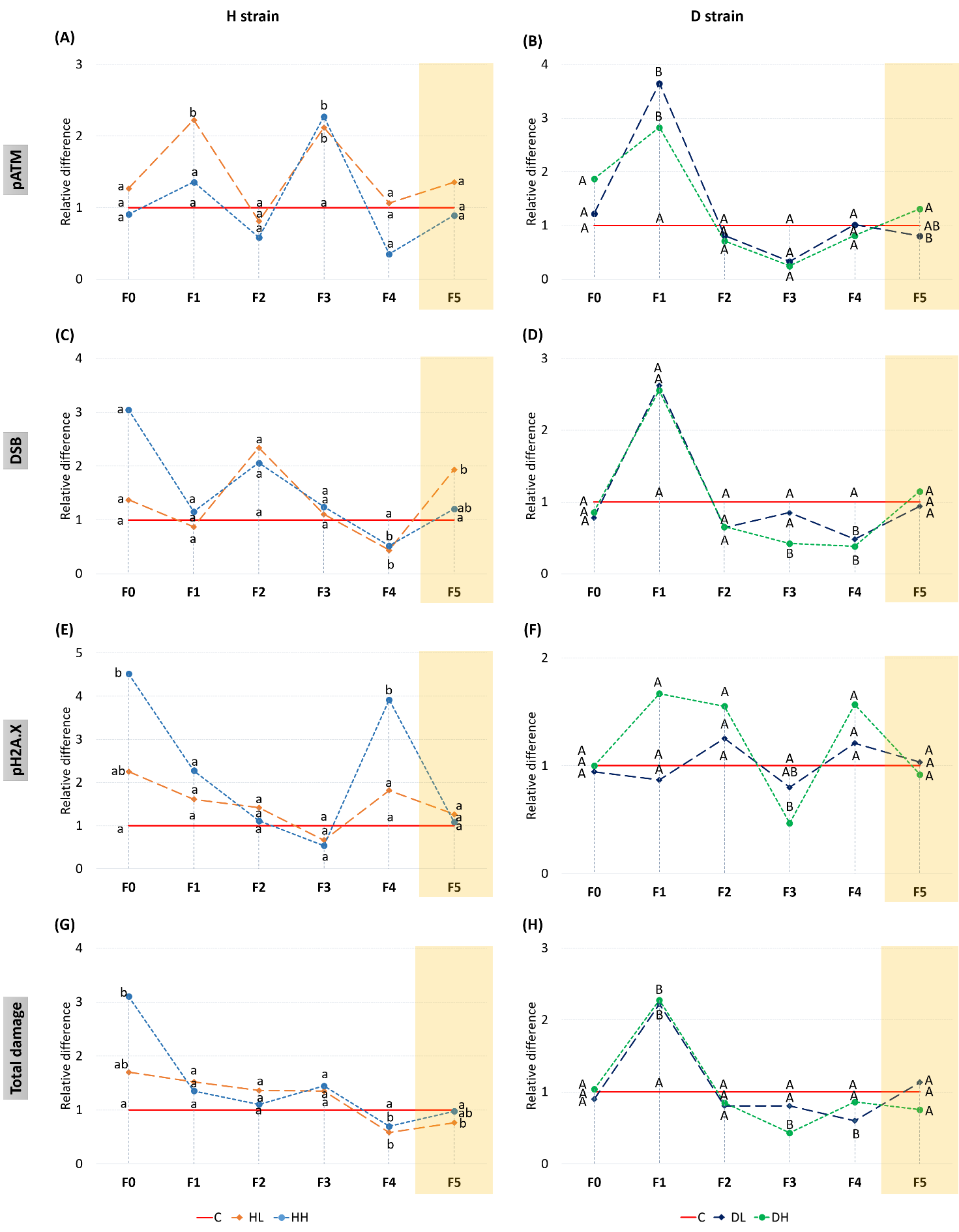
***

**Figure S1.** DNA damage measured in gut cells of wild (H) and long-lived strain (D) of *Acheta domesticus*; (A, B) pATM, (C, D) double-strand breaks, (E, F) pHA2.X, (G, H) total damage. Abbreviations: Generation 1-5 (F0-F4): (C) control animals fed uncontaminated food; (L) lower and (H) higher groups of animals fed GO-contaminated food at a concentration of 0.02 or 0.2 mg∙kg^–1^ of dry food, respectively; F5 – animals fed uncontaminated food. The average values are the relative differences in correspondence with controls for every generation and strain. Significant differences were measured using ANOVA (Fisher test; p < 0.05); letters denote differences among the experimental groups in strain.

**
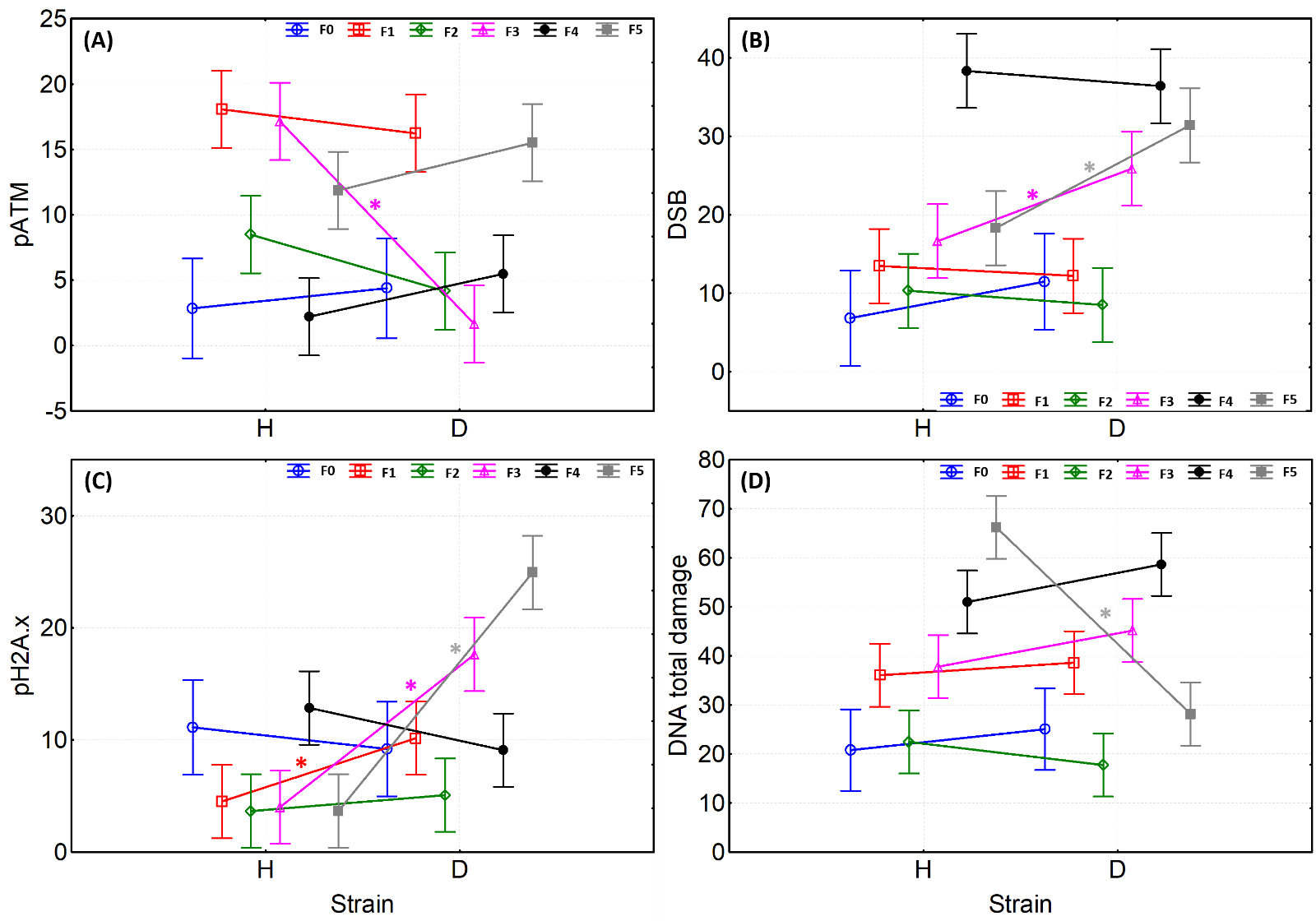
**

**Figure S2.** Differences between strains among generations in DNA damage measured in gut cells of wild (H) and long-lived strain (D) of *Acheta domesticus*; (A) pATM, (B) double-strand breaks, (C) pHA2.X, (D) total DNA damage. Abbreviations: six generations marked with different colors. Significant differences were measured using ANOVA, Expected Marginal Means (p < 0.05); Vertical bars indicate 0.95 confidence intervals; asterisks denote differences between strains among generations.


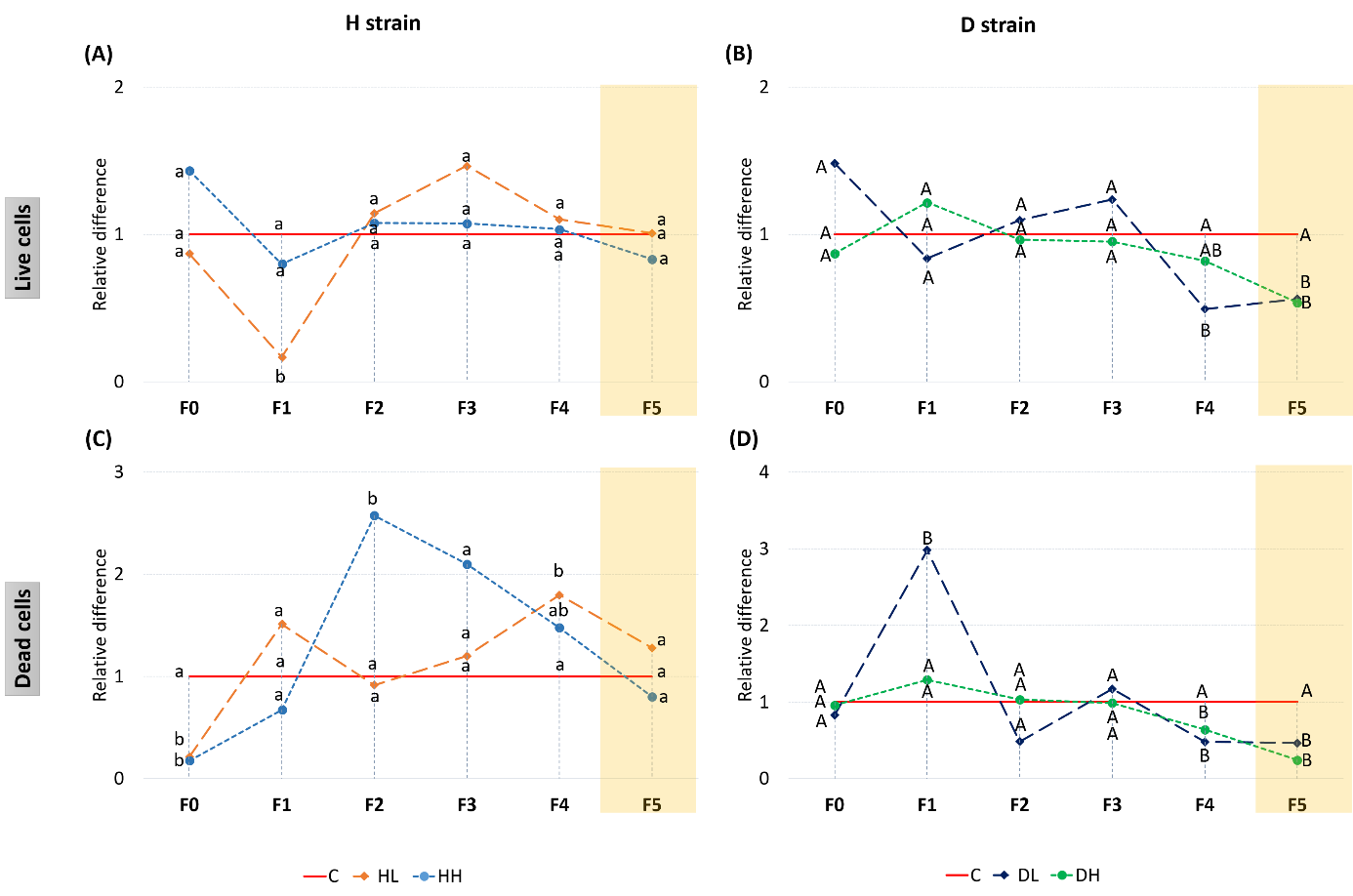


**Figure S3.** Live and dead cells were measured in gut cells of wild (H) and long-lived strain (D) of *Acheta domesticus*; (A, B) live cells, and (C, D) dead cells. Abbreviations: Generation 1-5 (F0-F4): (C) control animals fed uncontaminated food; (L) lower and (H) higher groups of animals fed GO-contaminated food at a concentration of 0.02 or 0.2 mg∙kg^–1^ of dry food, respectively; F5 – animals fed uncontaminated food. The average values are the relative differences in correspondence with controls for every generation and strain. Significant differences were measured using ANOVA (Fisher test; p < 0.05); letters denote differences among the experimental groups in strain.


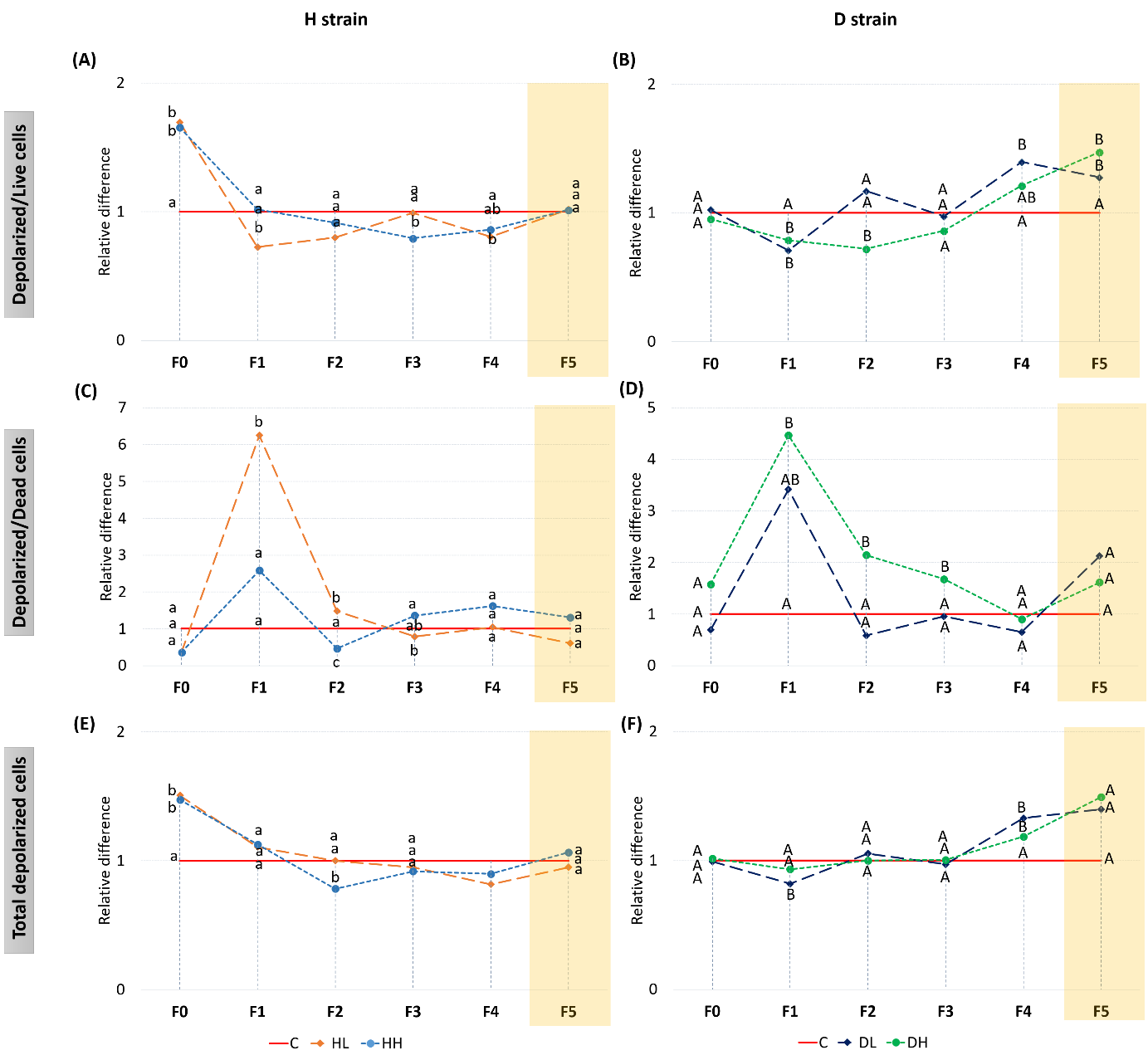


**Figure S4.** Mitopotential measured in gut cells of wild (H) and long-lived strain (D) of *Acheta domesticus*; (A, B) depolarized live cells, (C, D) depolarized dead cells, (E, F) total depolarized cells. Abbreviations: Generation 1-5 (F0-F4): (C) control animals fed uncontaminated food; (L) lower and (H) higher groups of animals fed GO-contaminated food at a concentration of 0.02 or 0.2 mg∙kg^–1^ of dry food, respectively; F5 – animals fed uncontaminated food. The average values are the relative differences in correspondence with controls for every generation and strain. Significant differences were measured using ANOVA (Fisher test; p < 0.05); letters denote differences among the experimental groups in strain.

**
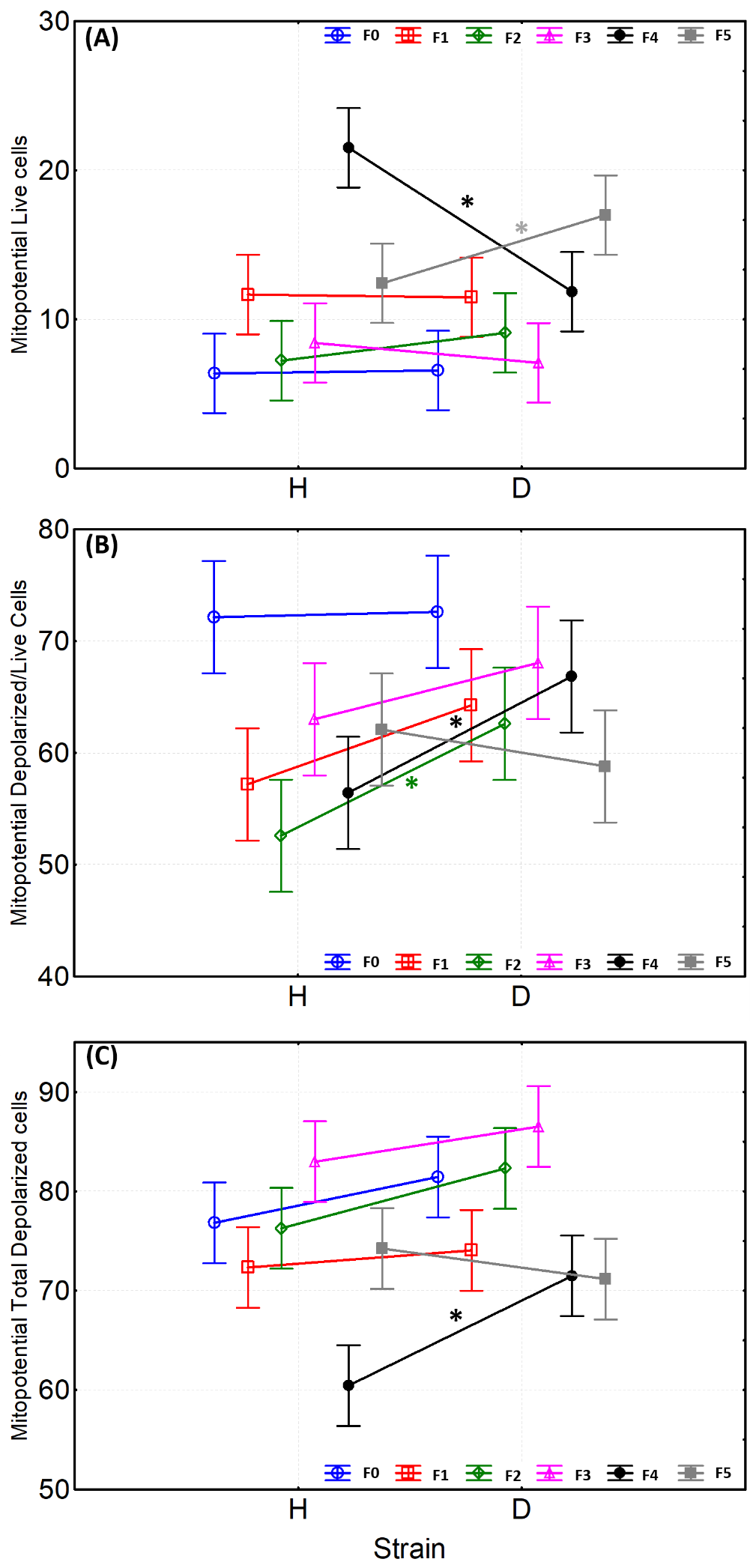
Figure S5.** Mitopotential differences between strains among generations in gut cells of wild (H) and long-lived strain (D) of *Acheta domesticus*; (A) Depolarized live cells, (B) Depolarized dead cells, (C) Total depolarized cells. Abbreviations: six generations marked with different colors. Significant differences were measured using ANOVA, Expected Marginal Means (p < 0.05); Vertical bars indicate 0.95 confidence intervals; asterisks denote differences between strains among generations.


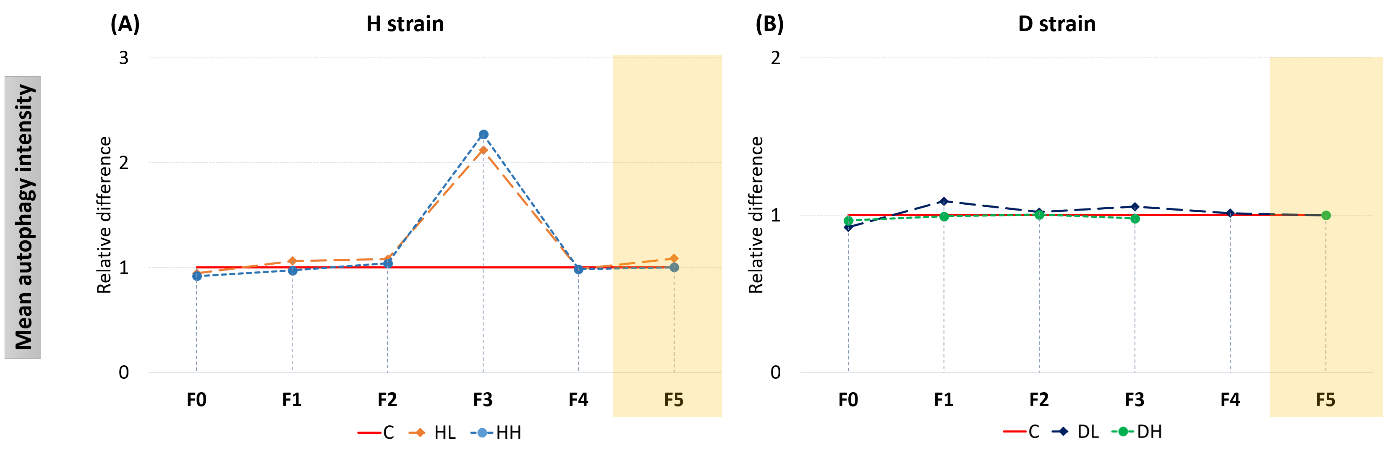


**Figure S6**. Mean autophagy intensity (A, B) measured in gut cells of wild (H) and long-lived strain (D) of *Acheta domesticus*; Abbreviations: Generation 1-5 (F0-F4): (C) control animals fed uncontaminated food; (L) lower and (H) higher groups of animals fed GO-contaminated food at a concentration of 0.02 or 0.2 mg∙kg^–1^ of dry food, respectively; F5 – animals fed uncontaminated food. F4, strain D – no data. The average values are the differences in correspondence with the reference value – the control for every generation and strain.


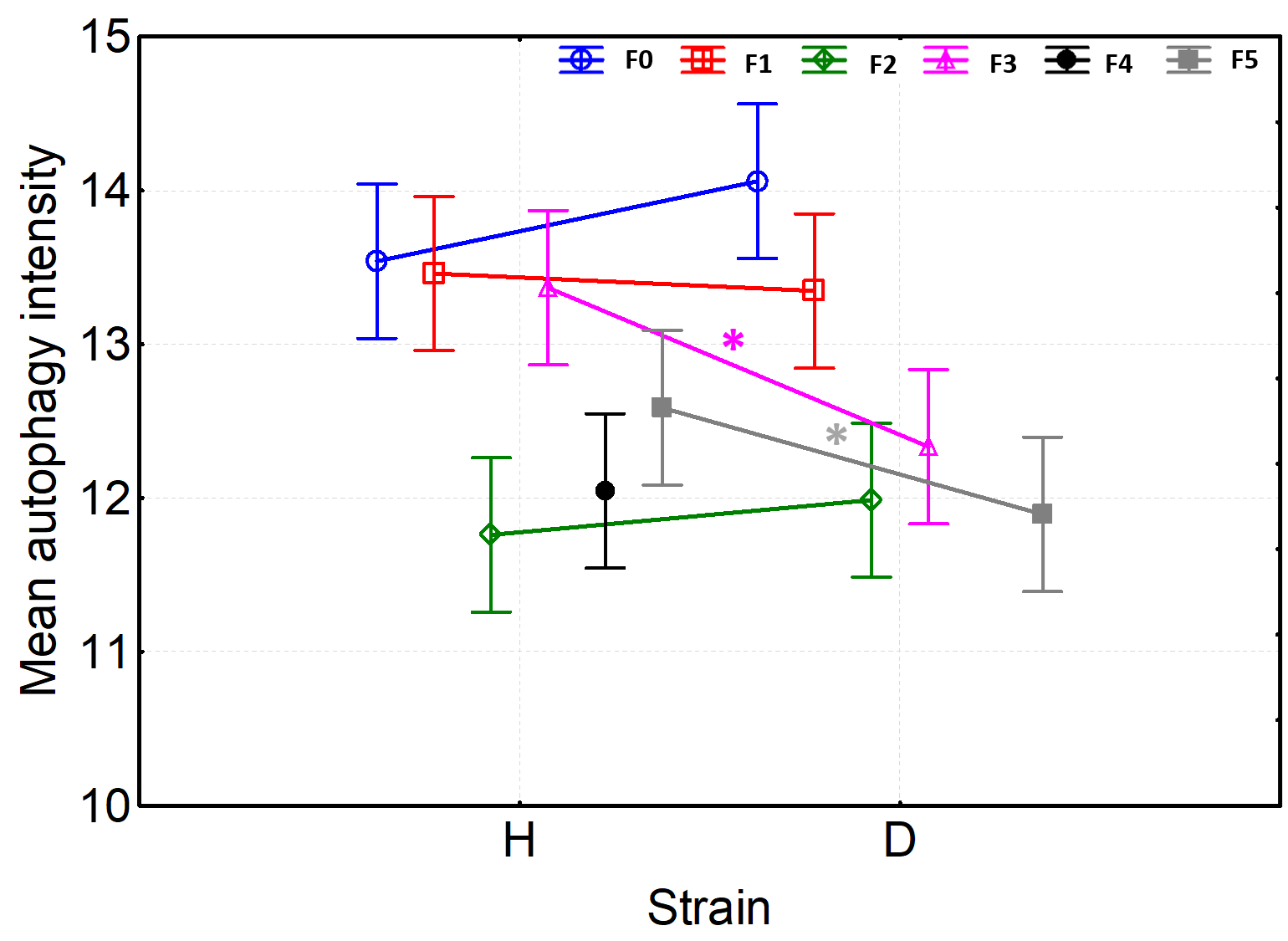


**Figure S7.** Differences between strains among generations in mean autophagy intensity in gut cells of wild (H) and long-lived strain (D) of *Acheta domesticus*. Abbreviations: six generations marked with different colors. Significant differences were measured using ANOVA, Expected Marginal Means (p < 0.05); Vertical bars indicate 0.95 confidence intervals; asterisks denote differences between strains among generations.

**
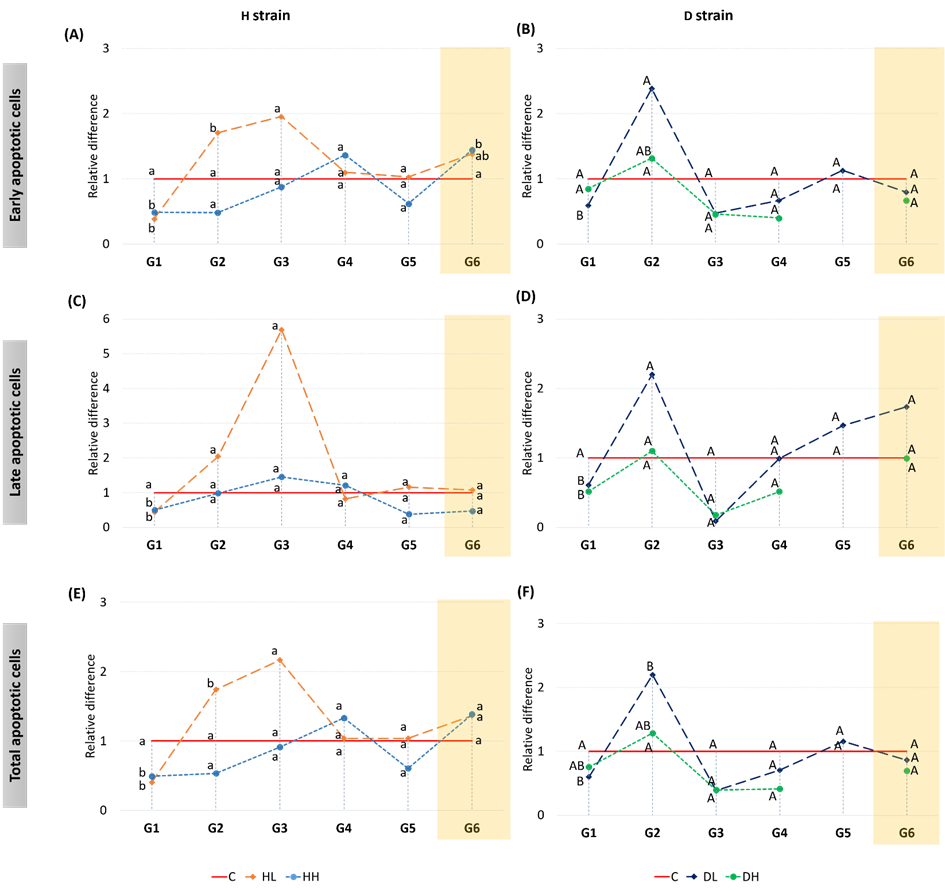
**

**Figure S8.** Apoptosis was measured in gut cells of wild (H) and long-lived strain (D) of *Acheta domesticus*: (A, B) early apoptotic cells (C, D), late apoptotic cells (E, F), total apoptotic cells. Abbreviations: Generation 1-5 (F0-F4): (C) control animals fed uncontaminated food; (L) lower and (H) higher groups of animals fed GO-contaminated food at a concentration of 0.02 or 0.2 mg∙kg^–1^ of dry food, respectively; F5 – animals fed uncontaminated food. F4, strain D – no data. The average values are the relative differences in correspondence with controls for every generation and strain. Significant differences were measured using ANOVA (Fisher test; p < 0.05); letters denote differences among the experimental groups in strain.

**
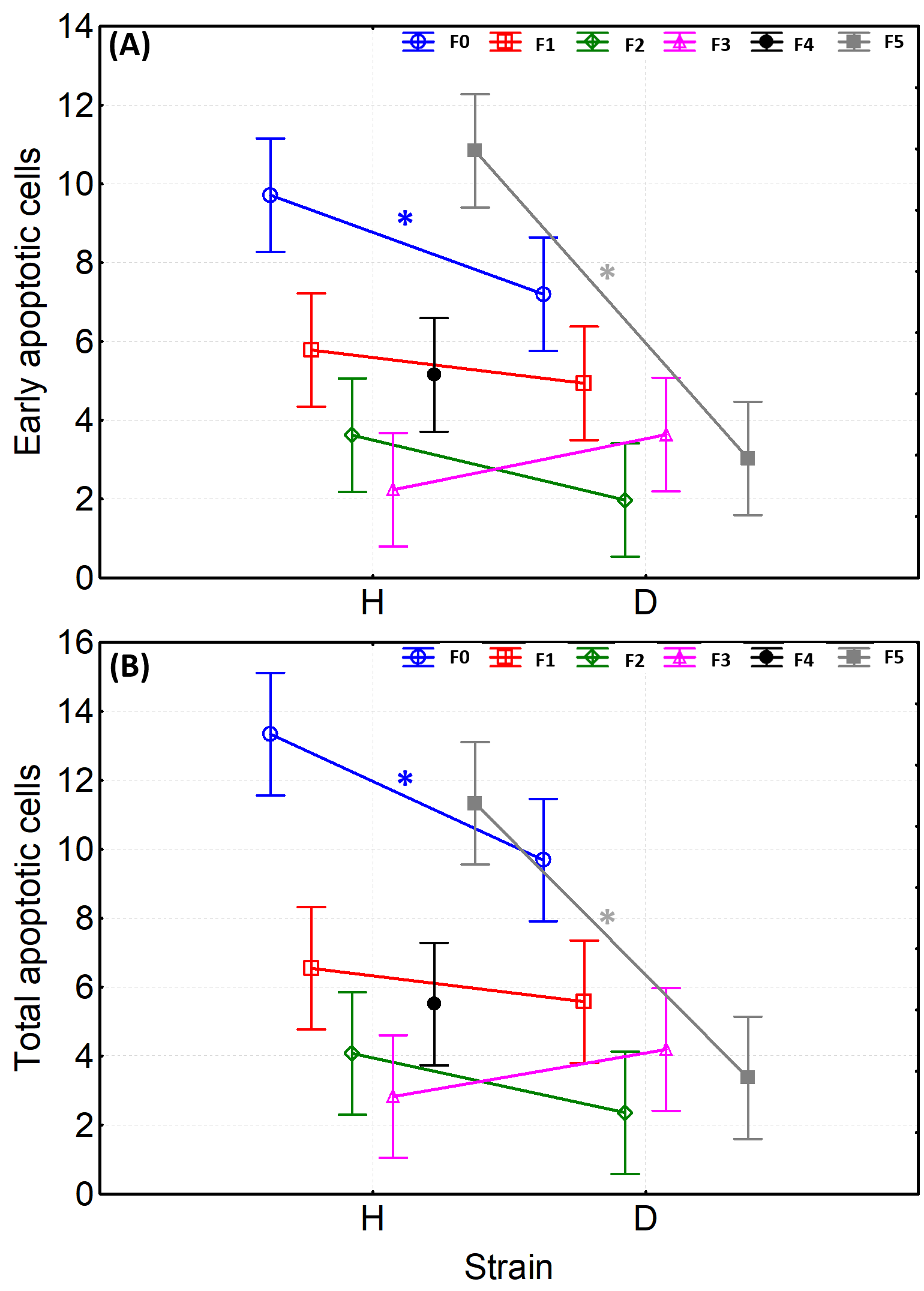
**

**Figure S9.** Differences between strains among generations in the share of apoptotic cells in gut cells of wild (H) and long-lived strain (D) of *Acheta domesticus*; (A) Early apoptotic cells, (B) Total apoptotic cells. Abbreviations: six generations marked with different colors. Significant differences were measured using ANOVA, Expected Marginal Means (p < 0.05); Vertical bars indicate 0.95 confidence intervals; asterisks denote differences between strains among generations.

**Table S5.** Mitopotential average and standard deviation values for investigated groups (C- control; L-low; H-high).

| *Mitopotential* | | | | | | | | | | | | |
| --- | --- | --- | --- | --- | --- | --- | --- | --- | --- | --- | --- | --- |
| Generation | Strain | Group | Depolarized/  Live [%] | | Depolarized/  Dead [%] | | Total depolarized [%] | | Live [%] | | Dead [%] | |
|  |  |  | Average | SD | Average | SD | Average | SD | Average | SD | Average | SD |
| F0 | H | C | 49.74 | 15.62 | 8.17 | 1.72 | 57.91 | 15.90 | 5.78 | 2.30 | 36.31 | 16.53 |
|  |  | L | 84.33 | 4.30 | 2.92 | 1.25 | 87.25 | 3.51 | 5.02 | 1.87 | 7.73 | 4.10 |
|  |  | H | 82.35 | 5.81 | 2.96 | 1.52 | 85.31 | 4.47 | 8.29 | 1.81 | 6.40 | 3.65 |
|  | D | C | 73.13 | 5.48 | 8.10 | 3.30 | 81.23 | 2.88 | 5.87 | 0.96 | 3.29 | 1.47 |
|  |  | L | 74.97 | 3.27 | 5.62 | 2.88 | 80.59 | 3.78 | 8.71 | 3.17 | 2.87 | 1.28 |
|  |  | H | 69.75 | 4.70 | 12.78 | 2.37 | 82.53 | 3.57 | 5.11 | 1.84 | 2.37 | 1.06 |
| F1 | H | C | 62.51 | 4.17 | 4.62 | 1.87 | 67.13 | 3.38 | 17.79 | 6.29 | 15.08 | 4.73 |
|  |  | L | 45.35 | 16.71 | 28.88 | 19.11 | 74.23 | 11.36 | 2.95 | 1.84 | 22.82 | 10.09 |
|  |  | H | 63.68 | 6.08 | 11.94 | 6.23 | 75.62 | 6.62 | 14.23 | 3.62 | 10.15 | 3.03 |
|  | D | C | 77.19 | 7.31 | 3.31 | 4.14 | 80.50 | 5.71 | 11.26 | 2.05 | 4.14 | 1.85 |
|  |  | L | 54.69 | 10.51 | 11.31 | 12.48 | 66.00 | 11.33 | 9.43 | 2.60 | 12.48 | 5.58 |
|  |  | H | 60.89 | 9.83 | 14.78 | 4.85 | 75.67 | 5.12 | 13.71 | 1.93 | 4.85 | 2.17 |
| F2 | H | C | 58.1 | 9.41 | 11.03 | 8.74 | 82.25 | 2.86 | 6.72 | 1.18 | 11.03 | 2.84 |
|  |  | L | 46.49 | 7.81 | 10.09 | 7.13 | 82.22 | 3.54 | 7.69 | 1.61 | 10.09 | 2.43 |
|  |  | H | 53.15 | 5.38 | 28.39 | 7.24 | 64.36 | 11.13 | 7.25 | 0.48 | 28.39 | 11.17 |
|  | D | C | 64.97 | 12.86 | 15.86 | 2.20 | 80.83 | 3.38 | 8.90 | 1.78 | 2.20 | 0.98 |
|  |  | L | 76.00 | 5.40 | 9.26 | 1.94 | 85.26 | 3.13 | 9.77 | 1.78 | 1.94 | 0.87 |
|  |  | H | 46.82 | 7.87 | 34.00 | 2.31 | 80.82 | 2.75 | 8.58 | 0.67 | 2.31 | 1.03 |
| F3 | H | C | 67.84 | 12.29 | 6.01 | 10.67 | 86.87 | 2.86 | 7.12 | 1.91 | 6.01 | 2.66 |
|  |  | L | 67.32 | 8.33 | 7.21 | 7.07 | 82.35 | 5.08 | 10.44 | 3.97 | 7.21 | 2.56 |
|  |  | H | 53.87 | 14.84 | 12.62 | 5.19 | 79.72 | 10.98 | 7.66 | 5.34 | 12.62 | 6.99 |
|  | D | C | 72.01 | 16.54 | 15.25 | 2.78 | 87.26 | 7.10 | 6.65 | 6.43 | 2.78 | 1.25 |
|  |  | L | 70.10 | 9.78 | 14.54 | 3.52 | 84.64 | 5.13 | 8.24 | 2.39 | 3.52 | 1.57 |
|  |  | H | 62.06 | 11.78 | 25.60 | 5.76 | 87.66 | 11.00 | 6.33 | 5.32 | 5.75 | 2.57 |
| F4 | H | C | 63.48 | 7.07 | 12.67 | 3.71 | 66.77 | 5.44 | 20.56 | 7.50 | 12.67 | 10.22 |
|  |  | L | 51.12 | 16.77 | 22.76 | 0.91 | 54.55 | 17.42 | 22.69 | 11.81 | 22.76 | 8.38 |
|  |  | H | 54.66 | 7.82 | 18.72 | 3.05 | 59.99 | 6.41 | 21.29 | 8.70 | 18.72 | 4.50 |
|  | D | C | 55.58 | 8.41 | 5.47 | 5.00 | 61.05 | 8.60 | 15.36 | 6.77 | 5.00 | 2.23 |
|  |  | L | 77.55 | 4.10 | 3.55 | 3.50 | 81.10 | 5.61 | 7.59 | 3.56 | 3.50 | 1.57 |
|  |  | H | 67.40 | 9.13 | 4.94 | 6.61 | 72.33 | 8.97 | 12.60 | 4.17 | 6.60 | 2.96 |
| F5 | H | C | 61.41 | 7.12 | 13.00 | 5.28 | 73.90 | 3.13 | 13.10 | 1.73 | 13.00 | 4.32 |
|  |  | L | 62.52 | 10.56 | 16.64 | 4.89 | 70.13 | 9.10 | 13.26 | 3.33 | 16.64 | 7.36 |
|  |  | H | 62.32 | 9.75 | 10.42 | 12.67 | 78.68 | 6.14 | 10.90 | 7.09 | 10.42 | 3.19 |
|  | D | C | 47.04 | 13.60 | 7.83 | 16.40 | 54.87 | 12.83 | 24.30 | 15.18 | 16.40 | 7.33 |
|  |  | L | 60.03 | 11.08 | 16.64 | 5.29 | 76.67 | 13.51 | 13.66 | 8.51 | 5.29 | 2.37 |
|  |  | H | 69.31 | 4.80 | 12.63 | 1.55 | 81.94 | 5.02 | 13.04 | 5.97 | 1.55 | 0.69 |

**Table S6.** Apoptosis average and standard deviation values for investigated groups (C- control; L-low; H-high).

| *Apoptosis* | | | | | | | | |
| --- | --- | --- | --- | --- | --- | --- | --- | --- |
| Generation | Strain | Group | Early  apoptotic [%] | | Late  apoptotic [%] | | Total  apoptotic [%] | |
|  |  |  | Average | SD | Average | SD | Average | SD |
| F0 | H | C | 15.54 | 9.27 | 5.63 | 3.27 | 21.17 | 12.29 |
|  |  | L | 6.02 | 1.28 | 2.45 | 1.35 | 8.47 | 1.01 |
|  |  | H | 7.58 | 1.72 | 2.80 | 2.66 | 10.38 | 4.21 |
|  | D | C | 8.83 | 5.12 | 3.51 | 2.25 | 12.34 | 7.20 |
|  |  | L | 5.26 | 0.80 | 2.14 | 1.33 | 7.40 | 1.50 |
|  |  | H | 7.51 | 2.20 | 1.81 | 0.61 | 9.31 | 2.63 |
| F1 | H | C | 5.43 | 1.72 | 0.57 | 0.49 | 6.00 | 1.72 |
|  |  | L | 9.28 | 3.93 | 1.16 | 1.12 | 10.44 | 4.95 |
|  |  | H | 2.63 | 0.57 | 0.56 | 0.77 | 3.19 | 0.95 |
|  | D | C | 3.15 | 0.01 | 0.58 | 0.61 | 3.73 | 1.60 |
|  |  | L | 7.51 | 1.20 | 1.28 | 0.15 | 8.20 | 2.35 |
|  |  | H | 4.15 | 1.33 | 0.64 | 0.34 | 4.79 | 1.45 |
| F2 | H | C | 2.83 | 0.73 | 0.17 | 0.10 | 3.00 | 0.75 |
|  |  | L | 5.54 | 1.80 | 0.96 | 0.48 | 6.49 | 2.19 |
|  |  | H | 2.49 | 0.66 | 0.24 | 0.07 | 2.73 | 0.67 |
|  | D | C | 3.06 | 2.48 | 0.91 | 1.38 | 3.97 | 3.82 |
|  |  | L | 1.45 | 0.79 | 0.08 | 0.09 | 1.53 | 0.76 |
|  |  | H | 1.41 | 0.51 | 0.16 | 0.14 | 1.57 | 0.55 |
| F3 | H | C | 1.93 | 0.76 | 0.58 | 0.60 | 2.52 | 1.24 |
|  |  | L | 2.12 | 0.64 | 0.48 | 0.18 | 2.61 | 0.81 |
|  |  | H | 2.64 | 0.51 | 0.71 | 0.27 | 3.35 | 0.73 |
|  | D | C | 5.27 | 2.60 | 0.66 | 0.19 | 5.93 | 2.64 |
|  |  | L | 3.53 | 1.27 | 0.66 | 0.66 | 4.18 | 1.89 |
|  |  | H | 2.11 | 0.48 | 0.34 | 0.20 | 2.45 | 0.45 |
| F4 | H | C | 5.83 | 2.32 | 0.42 | 0.41 | 6.26 | 2.62 |
|  |  | L | 5.99 | 2.89 | 0.49 | 0.48 | 6.48 | 3.35 |
|  |  | H | 3.62 | 0.78 | 0.16 | 0.11 | 3.78 | 0.85 |
|  | D | C | 6.55 | 1.05 | 0.51 | 0.37 | 7.06 | 1.34 |
|  |  | L | 7.40 | 0.47 | 0.75 | 0.20 | 8.14 | 0.55 |
|  |  | H | — | — | — | — | — | — |
| F5 | H | C | 8.49 | 1.80 | 0.58 | 0.51 | 9.07 | 2.21 |
|  |  | L | 11.75 | 4.87 | 0.63 | 0.40 | 12.37 | 5.19 |
|  |  | H | 12.29 | 8.32 | 0.27 | 0.18 | 12.56 | 8.18 |
|  | D | C | 3.68 | 0.51 | 0.27 | 0.11 | 3.95 | 0.59 |
|  |  | L | 2.94 | 0.52 | 0.47 | 0.25 | 3.41 | 0.33 |
|  |  | H | 2.47 | 0.51 | 0.27 | 0.14 | 2.74 | 0.53 |

**Table S7.** DNA damage average and standard deviation values for investigated groups (C- control; L-low; H-high).

| *DNA damage* | | | | | | | | | | |
| --- | --- | --- | --- | --- | --- | --- | --- | --- | --- | --- |
| Generation | Strain | Group | pATM [%] | | DSB [%] | | pH2A.X [%] | | Total damage [%] | |
|  |  |  | Average | SD | Average | SD | Average | SD | Average | SD |
| F0 | H | C | 2.68 | 0.45 | 3.77 | 3.64 | 4.29 | 1.73 | 10.74 | 5.59 |
|  |  | L | 3.40 | 1.24 | 5.19 | 0.99 | 9.67 | 2.28 | 18.26 | 2.40 |
|  |  | H | 2.43 | 0.67 | 11.49 | 8.08 | 19.39 | 10.69 | 33.32 | 19.05 |
|  | D | C | 3.21 | 0.96 | 13.01 | 7.82 | 9.37 | 6.67 | 25.58 | 15.43 |
|  |  | L | 3.93 | 3.00 | 10.20 | 6.35 | 8.84 | 4.11 | 22.97 | 11.40 |
|  |  | H | 6.00 | 6.36 | 11.20 | 8.69 | 9.36 | 4.22 | 26.56 | 7.95 |
| F1 | H | C | 11.83 | 5.09 | 13.34 | 2.20 | 2.77 | 1.43 | 27.93 | 4.02 |
|  |  | L | 26.31 | 6.82 | 11.65 | 2.60 | 4.46 | 2.34 | 42.42 | 6.84 |
|  |  | H | 16.06 | 9.58 | 15.42 | 7.54 | 6.29 | 3.39 | 37.78 | 10.12 |
|  | D | C | 6.51 | 3.41 | 5.93 | 1.72 | 8.62 | 2.46 | 21.06 | 2.65 |
|  |  | L | 23.76 | 7.98 | 15.56 | 3.27 | 7.48 | 2.57 | 46.80 | 9.10 |
|  |  | H | 18.41 | 13.95 | 15.16 | 8.36 | 14.37 | 14.33 | 47.94 | 9.26 |
| F2 | H | C | 10.62 | 7.42 | 5.73 | 5.26 | 3.10 | 1.48 | 19.45 | 11.36 |
|  |  | L | 8.62 | 4.29 | 13.41 | 5.73 | 4.39 | 1.91 | 26.42 | 8.57 |
|  |  | H | 6.21 | 3.97 | 11.80 | 9.61 | 3.44 | 2.65 | 21.45 | 15.57 |
|  | D | C | 4.92 | 1.84 | 11.09 | 7.55 | 4.00 | 1.21 | 20.02 | 9.19 |
|  |  | L | 4.02 | 2.08 | 7.13 | 5.54 | 5.01 | 1.72 | 16.16 | 7.02 |
|  |  | H | 3.51 | 1.65 | 7.29 | 4.26 | 6.20 | 2.30 | 17.00 | 5.34 |
| F3 | H | C | 9.54 | 5.98 | 14.88 | 6.10 | 5.47 | 2.11 | 29.90 | 10.35 |
|  |  | L | 20.21 | 12.64 | 16.49 | 8.08 | 3.62 | 2.43 | 40.33 | 15.69 |
|  |  | H | 21.57 | 10.51 | 18.54 | 12.19 | 2.94 | 1.28 | 43.15 | 20.59 |
|  | D | C | 3.14 | 3.62 | 34.13 | 10.91 | 23.34 | 23.47 | 60.60 | 17.91 |
|  |  | L | 1.04 | 0.50 | 29.05 | 17.36 | 18.62 | 8.98 | 48.71 | 20.98 |
|  |  | H | 0.78 | 0.59 | 14.44 | 10.76 | 10.91 | 6.58 | 26.13 | 17.56 |
| F4 | H | C | 2.73 | 0.78 | 58.62 | 14.40 | 5.73 | 3.06 | 67.07 | 12.86 |
|  |  | L | 2.90 | 2.21 | 25.82 | 16.42 | 10.41 | 3.03 | 39.13 | 17.92 |
|  |  | H | 0.96 | 0.59 | 30.56 | 15.74 | 22.43 | 4.94 | 46.73 | 18.34 |
|  | D | C | 5.81 | 2.67 | 58.51 | 8.01 | 7.22 | 1.75 | 71.58 | 7.38 |
|  |  | L | 5.91 | 3.89 | 28.26 | 12.69 | 8.72 | 6.77 | 42.88 | 18.28 |
|  |  | H | 4.72 | 3.03 | 22.44 | 9.44 | 11.30 | 8.81 | 61.53 | 12.16 |
| F5 | H | C | 10.96 | 8.10 | 13.24 | 7.31 | 3.27 | 1.61 | 72.53 | 13.73 |
|  |  | L | 14.84 | 6.44 | 26.68 | 11.44 | 4.13 | 1.08 | 55.34 | 14.97 |
|  |  | H | 9.79 | 3.22 | 15.98 | 7.52 | 3.53 | 2.30 | 70.69 | 7.23 |
|  | D | C | 14.93 | 6.78 | 30.47 | 12.60 | 25.36 | 9.58 | 29.24 | 11.00 |
|  |  | L | 12.02 | 4.42 | 28.67 | 7.19 | 26.18 | 5.92 | 33.13 | 6.84 |
|  |  | H | 19.58 | 2.83 | 35.09 | 3.96 | 23.30 | 1.81 | 22.03 | 5.22 |

**Table S8.** Autophagy average and standard deviation values for investigated groups (L-low; H-high) in comparison to the control.

| *Autophagy* | | | | |
| --- | --- | --- | --- | --- |
| Generation | Strain | Group | Mean autophagy intensity | |
|  |  |  | Average | SD |
| F0 | H | C | 14.20 (reference) | — |
|  |  | L | 13.40 | 0.49 |
|  |  | H | 13.02 | 0.94 |
|  | D | C | 14.60 (reference) | — |
|  |  | L | 13.48 | 0.44 |
|  |  | H | 14.10 | 1.30 |
| F1 | H | C | 13.30 (reference) | — |
|  |  | L | 14.14 | 0.74 |
|  |  | H | 12.94 | 0.74 |
|  | D | C | 13.00 (reference) | — |
|  |  | L | 14.14 | 0.21 |
|  |  | H | 12.90 | 0.67 |
| F2 | H | C | 11.30 (reference) | — |
|  |  | L | 12.22 | 0.20 |
|  |  | H | 11.76 | 0.47 |
|  | D | C | 11.90 (reference) | — |
|  |  | L | 12.12 | 0.26 |
|  |  | H | 11.94 | 0.32 |
| F3 | H | C | 13.50 (reference) | — |
|  |  | L | 13.30 | 1.13 |
|  |  | H | 13.30 | 1.13 |
|  | D | C | 12.20 (reference) | — |
|  |  | L | 12.86 | 0.48 |
|  |  | H | 11.94 | 0.38 |
| F4 | H | C | 11.70 (reference) | — |
|  |  | L | 12.72 | 0.58 |
|  |  | H | 11.72 | 0.51 |
|  | D | C | 13.50 (reference) | — |
|  |  | L | 13.66 | 0.62 |
|  |  | H | — | — |
| F5 | H | C | 12.30 (reference) | — |
|  |  | L | 12.88 | 0.41 |
|  |  | H | 12.58 | 0.45 |
|  | D | C | 11.90 (reference) | — |
|  |  | L | 11.88 | 0.22 |
|  |  | H | 11.90 | 0.29 |
